# Predicting which genes will respond to transcription factor perturbations

**DOI:** 10.1093/g3journal/jkac144

**Published:** 2022-06-06

**Authors:** Yiming Kang, Wooseok J Jung, Michael R Brent

**Affiliations:** Center for Genome Sciences and Systems Biology, Washington University School of Medicine, St. Louis, MO 63110, USA; Department of Computer Science and Engineering, Washington University, St. Louis, MO 63108, USA; Center for Genome Sciences and Systems Biology, Washington University School of Medicine, St. Louis, MO 63110, USA; Department of Computer Science and Engineering, Washington University, St. Louis, MO 63108, USA; Center for Genome Sciences and Systems Biology, Washington University School of Medicine, St. Louis, MO 63110, USA; Department of Computer Science and Engineering, Washington University, St. Louis, MO 63108, USA; Department of Genetics, Washington University School of Medicine, St. Louis, MO 63110, USA

**Keywords:** transcriptional regulation, machine learning, transcription factor perturbation, ChIP-Seq, histone marks

## Abstract

The ability to predict which genes will respond to the perturbation of a transcription factor serves as a benchmark for our systems-level understanding of transcriptional regulatory networks. In previous work, machine learning models have been trained to predict static gene expression *levels* in a biological sample by using data from the same or similar samples, including data on their transcription factor binding locations, histone marks, or DNA sequence. We report on a different challenge—training machine learning models to predict which genes will respond to the perturbation of a transcription factor *without using any data from the perturbed cells*. We find that existing transcription factor location data (ChIP-seq) from human cells have very little detectable utility for predicting which genes will respond to perturbation of a transcription factor. Features of genes, including their preperturbation expression level and expression variation, are very useful for predicting responses to perturbation of any transcription factor. This shows that some genes are poised to respond to transcription factor perturbations and others are resistant, shedding light on why it has been so difficult to predict responses from binding locations. Certain histone marks, including H3K4me1 and H3K4me3, have some predictive power when located downstream of the transcription start site. However, the predictive power of histone marks is much less than that of gene expression level and expression variation. Sequence-based or epigenetic properties of genes strongly influence their tendency to respond to direct transcription factor perturbations, partially explaining the oft-noted difficulty of predicting responsiveness from transcription factor binding location data. These molecular features are largely reflected in and summarized by the gene’s expression level and expression variation. Code is available at https://github.com/BrentLab/TFPertRespExplainer.

## Introduction

Understanding the function of a genome requires knowing which transcription factors (TFs) directly regulate each gene. A systems-level understanding should also enable us to predict which genes will change in expression level in response to direct perturbations of TFs. It was hoped that determining where in the genome each TF binds by chromatin-immunoprecipitation (ChIP) would go a long way toward solving these problems, but several studies have shown that the set of genes whose promoters are bound by a TF and the set of genes that respond to perturbations of that TF does not overlap much (Gitter *et al.* 2009; Lenstra and Holstege 2012; Cusanovich *et al.* 2014; Kang *et al.* 2020). Genes that are responsive but not bound may be indirect targets of the TF. Genes that are not responsive despite the fact that their regulatory DNA is bound by the perturbed TF constitute a greater mystery. Currently, we cannot predict which bound genes will respond to a perturbation and which will not. In this article, we take on the challenge of predicting whether a gene will respond to perturbation of a TF by using data on where the TF binds along with a variety of gene-centric features that do not depend on which TF is perturbed, including histone marks (HMs), chromatin accessibility, dinucleotide frequencies, and the gene’s preperturbation expression level and expression variation.

A number of studies have shown success in predicting the expression *levels* of genes by using TF binding signals (Middendorf *et al.* 2004; Ouyang *et al.* 2009; Schmidt *et al.* 2017) or HMs in each gene’s regulatory region (Karlić *et al.* 2010; Cheng *et al.* 2011; Dong *et al.* 2012; McLeay *et al.* 2012; Singh *et al.* 2016; Read *et al.* 2019). These models predict expression level in a given sample by using data from the same cell type and similar growth conditions. As a result, the epigenetic features used for prediction could be causes, consequences, or merely correlates of gene expression level (Henikoff and Shilatifard 2011). Recently, deep neural networks have been used to predict the expression level of a gene from the DNA sequence flanking it (Kelley *et al.* 2018; Zhou *et al.* 2018; Washburn *et al.* 2019; Agarwal and Shendure 2020). Models have also been trained to predict the variability of gene expression within or across cell types (Ouyang *et al.* 2009; Zhou *et al.* 2014; González *et al.* 2015; Crow *et al.* 2019; Sigalova *et al.* 2020). In addition to the above genomic features, combining binding sites of RNA-binding proteins and microRNAs in transcripts with TF binding sites at promoters was also found to be predictive (Tasaki *et al.* 2020).

We have taken on a different challenge—training machine learning models that can predict which genes will respond to the perturbation of a TF without using any data from perturbed cells. More precisely, the models predict the probability that a given gene will respond to the perturbation of a given TF. Because the predictive features are measured in unperturbed cells, they cannot be consequences of the perturbation or the response. Models are trained using perturbation-response data for a subset of TFs and tested on different TFs, holding out the possibility of using predicted perturbation responses when perturbation-response experiments are not practical. The overall accuracy of the models serves as a benchmark for our understanding of global regulatory networks. Perhaps more importantly, analysis of how the trained models make predictions on unseen TFs provides insight into the factors that determine which genes respond to a TF perturbation. Many methods have been developed to explain how specific features and feature values influence a complex model’s predictions (Breiman 2001; Zeiler and Fergus 2012; Zhou and Troyanskaya 2015; Fisher *et al.* 2019; Molnar 2019). In this article, we rely on SHAP values (Lundberg and Lee 2017; Lundberg *et al.* 2018). A SHAP value represents the influence of one feature on one predicted probability. The SHAP values for a prediction allocate to each feature its share of responsibility for the deviation of that prediction from the default prediction that would be made in the absence of any information from features. The default is the fraction of positive examples among all training instances (i.e. the expected value of the label). The sum of the SHAP values for a prediction is equal to the deviation of the prediction from the default [See *Methods* and ref. (Lundberg *et al.* 2018) for details]. A feature with a positive SHAP value pushes the model to predict a higher probability of response for the gene in question, while a feature with a negative SHAP value pushes the model to predict a lower probability of response. The magnitude of the SHAP value of a feature of one prediction indicates how much that feature influences that prediction.

SHAP analysis, complemented by analyses of model accuracy, provides several surprising biological and methodological insights.

1. Existing genome-scale data on TF binding locations in human cells, including ENCODE data on K562 cells, have little value for predicting which genes will respond to perturbation of a TF. However, yeast data obtained by newer methods (transposon calling cards or ChIP-exo) are useful.
2. A few HMs have value for predicting perturbation responses, primarily when they occur in the gene body downstream of the transcription start site (TSS).
3. For both yeast and human, preperturbation gene expression level and gene expression variation (GEX features) were useful for predicting whether a gene would respond to perturbation of any TF; for human cells, they were far and away from the most useful features. When these features are available, HMs provide no additional information that is useful for predicting perturbation responses in human cells.

In summary, the properties of the gene itself have a major influence on its tendency to respond to regulatory perturbations, regardless of which TF is perturbed. The extent to which this tendency is determined by the gene’s epigenetic state or its inherent properties remains to be seen.

## Methods

### TF-perturbation response data

For yeast cells, we downloaded microarray data taken 15 min after inducing overexpression of 194 TFs from https://idea.research.calicolabs.com/data (Hackett *et al.* 2020). We previously showed that the responses at the 15-min timepoint have the best correspondence with binding location (Kang *et al.* 2020). Column *log2_shrunken_timecourses* from file “Raw & processed gene expression data” was used. Since these values were already shrunken toward zero, any gene with a nonzero value was defined as responsive. For human K562 cells, we used all RNA-seq expression profiles after gene perturbation from the ENCODE database (Dunham *et al.* 2012; Davis *et al.* 2018; Moore *et al.* 2020). Perturbations include disabling mutations introduced by CRISPR, CRISPR inference, small-interfering RNA, and small-hairpin RNA. We downloaded RSEM expected counts of TF-perturbation and control profiles. For each of the 355 experiments, we ran DESeq2 (V1.10.1; Love *et al.* 2014) to identify differentially expressed genes by comparing the TF-perturbation replicates to the corresponding control replicates. Genes with adjusted *P < *0.05 and absolute log2 fold-change > 0.5 were considered responsive. For human H1 (hESC) cells, we used all RNA-seq reads downloaded from NCBI bioproject PRJDB5361 (Nakatake *et al.* 2020). We ran Nextflow’s nf-core/rnaseq to get RSEM expected counts for TF-perturbation and control samples for which we had binding data. We ran DESeq2 (Love *et al.* 2014) to identify differentially expressed genes by comparing the TF-perturbation replicates to the corresponding control replicates. Genes with adjusted *P < *0.05 and absolute log2 fold-change > 0.5 were considered responsive. For human HEK293 cells, we used all overexpression profiles downloaded from NCBI GEO Series GSE76495 (Schmitges *et al.* 2016; file GSE76495_OE .vsd_normalized.log2.txt.gz). Since there were no replicates and hence no statistical significance, genes with absolute log2 fold-change >0.5 were considered responsive.

### Preperturbation gene expression features

The preperturbation expression level feature of a gene is its median expression level across all samples measured prior to the TF perturbation. For K562 and H1 data (RNA-Seq data), we used the median log TPM levels of all control replicates. For HEK293 data, we calculated the median log TPM levels of all wild-type replicates from NCBI GEO Series GSE122425 (Sun *et al.* 2019). For yeast (microarray data), we used log fluorescence levels of the red (experimental) channel measured at time 0 (before TF induction). To construct a gene expression variation feature that is independent of the expression level, we used the method of Sigalova *et al.* (2020). Briefly, LOESS regression was performed producing a model that predicts the coefficient of variation of each gene as a function of its median expression level and the residuals were used as the expression variation feature (see Supplementary Fig. 2).

### TF binding location features

All coordinate-dependent features were mapped to yeast genome build sacCer3 or human build GRCh38. Yeast binding location data from transposon calling cards (16 TFs) was obtained from publications (Wang *et al.* 2011; Shively *et al.* 2019; Kang *et al.* 2020), which reported a target gene to be significantly bound if *P* ≤ 0.001. Binding locations from ChIP-exo were obtained directly from the authors of publications (20 TFs; Bergenholm *et al.* 2018; Holland *et al.* 2019), who called a peak significant if it had signal-to-noise ratio > 2. We lifted over the peak locations reported for strain CEN.PK to sacCer3 coordinates (strain S288C; see Supplementary Methods). For human cells, we downloaded ChIP-seq peaks for 42 TFs in K562 cells and 23 TFs in H1 cells from ENCODE. We consider a gene to be a TF only if it has a well-defined DNA-binding domain (Lambert *et al.* 2018). We used the “conservative” peaks, which have an irreproducible discovery rate ≤2%. The log10 *q*-value reported for each peak was used as the binding signal feature. For HEK293 cells, we downloaded the ChIP-seq peaks from NCBI GEO series GSE76494 (file GSE76494_combined_summits.motif_hits.per_protein.hg19.tar.gz; Schmitges *et al.* 2016).

### Histone modifications and chromatin accessibility data

For yeast, we used histone modification data at timepoint 0 (in YPD batch cultures prior to addition of a diamide stress) from ref. Weiner *et al.* (2015), which was produced using MNase-ChIP-Seq (GEO accession GSE61888). We used chromatin accessibility data at timepoint 0 (prior to addition of osmotic stress) from ref. Schep *et al.* (2015; GSE66386). For each of the 3 human cell lines, we downloaded the coverage data (fold-change over control) for histone modifications and chromatin accessibility from ENCODE. We supplemented the coverage data for H3K27me3 in HEK293 cells using NCBI GEO dataset GSM3907592 (Lamb *et al.* 2019) due to its unavailability in ENCODE.

### Mapping genome-wide features to cis-regulatory regions

We defined yeast promoter regions as (−1,000 bp, + 500 bp) relative to TSS. TSS coordinates were obtained from ref. de Boer *et al.* (2020). Inputs for each feature were summed within each of fifteen 100-bp bins to create 15 features. Human TSS coordinates were downloaded from Ensembl Release 92 (Cunningham *et al.* 2019). For each gene, we defined the *5′* *promoter* to be 4 kb centered on the 5′-end TSS. We define *alternative promoters* to be 4 kb regions centered at any TSS that is more than 2 kb from the 5′-end TSS and *enhancers* to be enhancers that are linked to the gene in GeneHancer V4.8 (Fishilevich *et al.* 2017). We used only “double elite” enhancers, whose existence and linkage to the target gene are both supported by at least 2 types of evidence. This includes 96,008 enhancer–promoter pairs.

Each gene must have an equal number of features to create a rectangular feature matrix, even though genes differ in how many alternative TSSs and enhancers they have. Alternative TSSs within 2 kb of the 5′ end were deemed to be included in the 5′ promoter and those further than 2 kb were treated as enhancers, since enhancers and promoters share most properties and functions (Andersson and Sandelin 2020). Signals located in enhancers were aggregated into features in 2 ways. The first method (*Prom + bin enhan*; Supplementary Fig. 3, blue) sums signals from enhancers that fall within each of 32 bins upstream of the 5′-end promoter region and another 32 bins downstream. The width of the bin closest to the TSS was 1 kb and each subsequent bin was larger by 1 kb (1, 2, 3 kb, etc.). Together with the 5′-end promoter, these bins include enhancers within 500 kb on either side of the 5′ TSS. The second method (*Prom + agg enhan*, Supplementary Fig. 3, green) sums signals that fall within 500 kb upstream or downstream of the promoter into 2 features. Only signals within defined enhancers were used.

### Training and testing machine learning algorithms

For each cell type, we trained and tested a model for predicting whether a gene will respond to the perturbation of TFs by cross-validating across all TFs. Each instance is a pair of protein-coding gene and TF. In a cross-validation fold, 90% of the TFs were selected at random; instances involving the remaining TFs were held out for testing. We trained the models used gradient-boosted trees implemented in XGBoost library (V 0.90; Chen and Guestrin 2016). See Supplementary Methods for details.

### Using SHAP to quantify the influences of features on predictions

SHAP values were calculated by the TreeExplainer function for XGBoost (Lundberg and Lee 2017; Lundberg *et al.* 2018).

### Calculating the *P*-values using random permutations

We trained models on randomly permuted data as described in the main text: 35 permutations each for Calling Cards, ChIP-exo, H1, and K562 datasets and 5 for HEK293 dataset due to time and memory constraints. For each permuted dataset, we carried out cross-validation training and testing in the same manner as we did for the real datasets and then calculated the mean and standard deviation of the AUPRC across permutations for each TF in each dataset. For each dataset, we then plotted the standard deviations against the random expectations for the same TFs on a log–log scale (Supplementary Fig. 6). These showed a clear linear trend for all TFs with at least 1% of genes responding to the perturbation, so we fit a linear model to the log standard deviation (LSD) as a function of log fraction of genes responding. For the human TFs with at least 1% of genes responding, we used the model-based prediction of the LSD for each TF to construct a normal null distribution around the expected AUPRC and calculated a *P*-value for the AUPRC obtained from the authentic, nonpermuted data. Because of relatively high variance around the predicted LSD for HEK293, we used more conservative LSD estimates based on a line fit to only the highest LSD TF in each quintile of percent responsive genes (Supplementary Fig. 6e). For TFs with less than 1% of genes responding, we used the actual SD on the permuted datasets rather than the model-based prediction.

## Results

### Modeling frameworks, features, and datasets

**Fig. 1.**
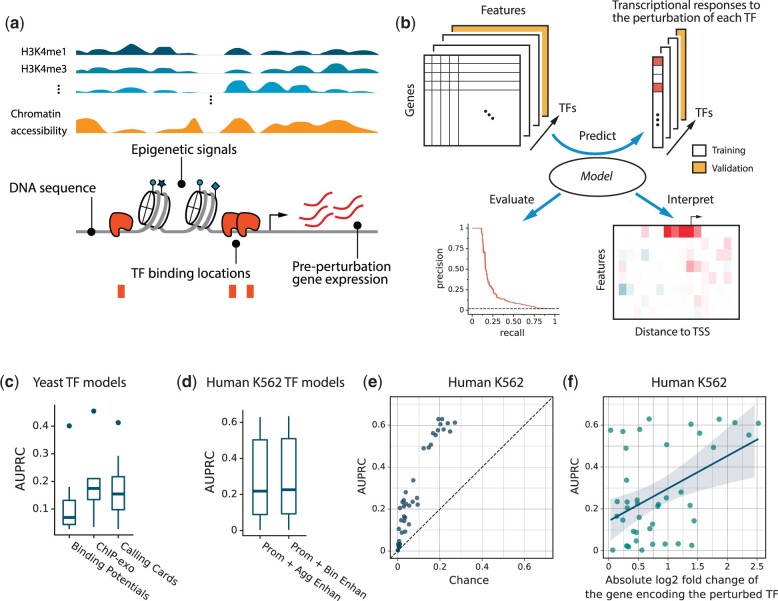
Approach and model and performance. a) Features for predicting transcriptional responses to TF perturbation. b) Framework for predicting responses, evaluating model performance, and estimating local feature influences. c) Accuracy comparison of yeast models trained with binding potential calculated from binding specificity models, binding location data from ChIP-exo experiments, or binding location data from calling cards experiments. Boxes show the distributions of AUPRC across 8 TFs for which all 3 data types are available. d) Model accuracy on human K562 cells using 2 methods of aggregating data from enhancers associated with each gene. e) Model accuracy in human K562 cells compared to chance. AUPRC for responses to perturbation of each TF is plotted against random expectation, which is the number of genes that respond to the perturbation divided by the total number of genes. f) Model accuracy in human K562 cells compared to the efficacy of the TF perturbation. AUPRC for responses to perturbation of each TF is plotted against the absolute value of the log2 fold-change of the gene encoding the perturbed TF.

We took a 2-step approach to understanding the determinants of transcriptional responses to TF perturbations: (1) train machine learning models to predict whether each gene will respond to a perturbation of a particular TF and (2) analyze the trained models to identify which genomic features they used to make their predictions on unseen data. We provided the models with 3 types of genomic features (Fig. 1a). First, data on the binding locations of the perturbed TF (location features). Second, data on the median and variance of each gene’s expression levels in unperturbed samples (GEX features). Third, data on each gene’s epigenomic context, including DNA accessibility and selected histone modifications (epigenetic features). We focused on 8 HMs that were previously shown to be most useful for predicting gene expression level (Karlić *et al.* 2010; Zhou *et al.* 2014; González *et al.* 2015; Kundaje *et al.* 2015; Singh *et al.* 2016). GEX and epigenetic features are gene-centric—they do not change as a function of which TF is perturbed. Only the binding location features are matched to the perturbed TF. We also provided the model with dinucleotide frequencies in the gene’s regulatory DNA. These frequencies are correlated with many other features, so if a model is using them, we wanted it to do so explicitly, rather than through their correlations with other features.

To generate a feature matrix, we defined cis-regulatory regions for each gene and mapped genomic data to them. For yeast genes, we assumed a regulatory region ranging from 1,000 bp upstream of the TSS to 500 bp downstream. Although most studies assume the yeast promoter is smaller than this, we expected that the models would learn which parts of this region are most predictive. For human genes, we included both proximal promoters and distal enhancers. The enhancer locations and target genes were taken from GeneHancer V4.8 (Fishilevich *et al.* 2017). This includes 96,008 enhancer–promoter pairs. Enhancers can be up to 500 kb to either side of the TSS. Human proximal promoters were defined as 2 kb upstream and downstream of the 5′-most TSS. This region contains most TSSs that are transcribed at any appreciable level (Supplementary Fig. 1). Alternative promoters outside of 5′-most proximal promoter were treated as additional enhancers (Andersson and Sandelin 2020).

To test whether certain locations within a regulatory region are more important than others, we divided the promoter regions into 100 bp subregions, each with its own features. Within each 100 bp subregion, signals from each of the assays for TF binding location, DNA accessibility, HMs, or dinucleotide frequencies were aggregated and discretized. For yeast, we used TF binding location data generated by 2 in vivo assays: transposon calling cards (Wang *et al.* 2011; Shively *et al.* 2019; Kang *et al.* 2020) and ChIP-exo (Bergenholm *et al.* 2018; Rossi *et al.* 2018). We showed previously that these datasets predict perturbation responses much better than older ChIP-chip data (Kang *et al.* 2020). We used data on yeast HMs from ref. Weiner *et al.* (2015) and chromatin accessibility from ref. Schep *et al.* (2015). For human models, we focused primarily on the K562 cell line because it has the most TFs that were ChIPped and perturbed in the ENCODE Project (Dunham *et al.* 2012; Davis *et al.* 2018; Moore *et al.* 2020). See Table 1 for a full list of data resources used. For both yeast and human, preperturbation expression variance was adjusted to make it independent of expression level (Sigalova *et al.* 2020; *Methods*; Supplementary Fig. 2).

**Table 1.** Data resources and statistics.

	Yeast S288C	Human K562	Human H1	Human HEK293
GEX after TF perturbation	Hackett *et al.* (2020)	ENCODE	Nakatake *et al.* (2020)	Schmitges *et al.* (2016)
TF binding [calling cards (CC)]	Wang *et al.* (2011), Shively *et al.* (2019), and Kang *et al.* (2020)	—	—	—
TF binding (ChIP-exo)	Bergenholm *et al.* (2018) and Rossi *et al.* (2018)	—	—	—
TF binding (ChIP-seq)	—	ENCODE	ENCODE	Schmitges *et al.* (2016)
Histone modifications	Weiner *et al.* (2015)	ENCODE	ENCODE	ENCODE; Lamb *et al.* (2019)
Chromatin accessibility	Schep *et al.* (2015)	ENCODE	ENCODE	ENCODE
Unperturbed GEX	Hackett *et al.* (2020)	ENCODE	Nakatake *et al.* (2020)	Sun *et al.* (2019)
Type of TF perturbation (time before harvest)	Overexpression (15 min)	Knockout/knockdown (3–6 d)	Overexpression (48 h)	Overexpression (24 h)
Cell state for TF binding	Unperturbed	Unperturbed	Unperturbed	TF overexpressed
Number of TFs	14 (CC)	42	23	80
	19 (ChIP)			
Median response rate	0.035 (CC)	0.040	0.020	0.070
	0.020 (ChIP)			
SD of response rate	0.120 (CC)	0.084	0.093	0.051
	0.073 (ChIP)			

ENCODE (Dunham *et al.* 2012; Davis *et al.* 2018; Moore *et al.* 2020).

We trained models to predict the probability that a gene will respond to a TF perturbation (Fig. 1b). For yeast, responsiveness was determined by using data from ref. Hackett *et al.* (2020), who measured transcriptional responses shortly after chemically inducing overexpression of each TF. For human, responsiveness was determined by using RNA-Seq after TF knockdown/knockout (K562) or overexpression (H1 and HEK293). All classification models were trained by using 10-fold cross-validation on TFs. That is, predictions for each TF were made by models that had not seen any binding or perturbation response data on that TF during training. Thus, they could be used to predict the outcome of a perturbation-response experiment on a TF before that experiment is carried out. (Cross-validation by gene yielded very similar prediction accuracy, Supplementary Fig. 13.) For yeast datasets, each training fold contained at least 77,000 instances; for human, at least 392,000 instances. For each dataset, the median percentage of genes that responded to a TF perturbation was between 2% and 7% with wide variation from TF to TF (Table 1).

We trained and tested models based on gradient-boosted trees by using XGBoost (Chen and Guestrin 2016), a machine learning algorithm that is considered state-of-the-art for this type of learning problem. Below, we analyze how the features influence the prediction of whether a gene will respond to the perturbation of a TF. We used precision-recall curves for accuracy evaluation and the area under the curve (AUPRC) as a summary statistic. This approach is appropriate because only a small fraction of genes is responsive to each perturbation, creating large class imbalances. The random expectation for AUPRC for responses to a given TF is the fraction of all genes that are responsive to that TF. This random expectation varies widely among TFs.

First, we tested classifiers using yeast TF binding location data from either transposon calling cards or ChIP-exo experiments, keeping all other features unchanged (Fig. 1c). We also tried replacing binding location data with TF binding potentials obtained by scanning a binding specificity model for the perturbed TF (Grant *et al.* 2011; Spivak and Stormo 2012) over promoter sequences. The binding potential was least useful, even when data on chromatin accessibility were also included in the model (Fig. 1c, Supplementary Fig. 3a).

Using XGBoost on the K562 ENCODE data (Table 1), we investigated 2 ways of incorporating binding and epigenetic features from enhancers. The 2 methods divide the region around the promoter into subregions in different ways and sum the signals from enhancers within each subregion to form a single feature value. The first method (*bin enhan*) sums signals over enhancers within subregions whose widths increase with their distance from the TSS (Supplementary Fig. 4, *Methods*). The second approach (*agg enhan*) sums signals from all enhancers upstream of the TSS to create one feature and all enhancers downstream of the TSS to create another. Models trained using the 2 strategies of enhancer-feature mapping show no significant difference in accuracy (*P* = 0.63, paired *t*-test; Fig. 1d, Supplementary Fig. 5), so we used the less numerous aggregated enhancer features in the remainder of the study.

Explaining what an algorithm learned is only interesting if it learned something significant. To test whether the predictions of the models were significantly better than would be expected by chance, we created artificial datasets for each TF in which the assignment of “responsive” or “nonresponsive” labels to genes was randomly permuted. Importantly, the number of responsive genes for each TF was the same as in the real data. We then retrained the models using the same, unmodified features for each gene and the same cross-validation procedure. As expected, the average AUPRC for each TF in randomized data was very close to the fraction of genes that responded to perturbation of that TF. For the human data, the log standard deviations of the AUPRCs for permuted data increased approximately linearly with the mean AUPRC for the same TF (Supplementary Fig. 6). We then calculated a *P*-value for the null hypothesis that the AUPRC obtained for a given TF from the true data was no better than the AUPRC obtained from the permutated data (see Supplementary Methods for details). Nearly, all TFs showed extremely small *P*-values indicating that the model trained on real data was better than random on that TF (Supplementary Files 1–5). At a threshold of *P* < 10^−3^, 143 of the 145 TFs in human datasets were significant as were all TFs in the yeast calling cards dataset. The yeast ChIP-exo data were the exception with only 12 of 19 TFs being significant at *P* < 10^−3^. For both yeast and human, all nonsignificant TFs were excluded from all future analyses.

When compared to random expectation, the accuracy on all remaining TFs was quite respectable. For the vast majority of TFs, the actual AUPRC was more than twice the random expectation (Fig. 1e, Supplementary Fig. 3, Supplementary Files 1–5). The K562 model achieved at least twice the random expectation for every TF, and for several TFs its actual performance was more than 10 times the random expectation (Supplementary File 3). In the K562 data, a major factor influencing accuracy was the effectiveness of the TF perturbation (Fig. 1f). Prediction accuracy was relatively good for all samples in which the absolute log2 fold-change of the TF in the perturbed sample relative to the unperturbed is greater than 1.5. However, this relationship did not hold for the other datasets, where TFs were perturbed by overexpression and most perturbations were large (Supplementary Fig. 7).

### The TF binding signal is useful for response prediction in yeast

The next step in our analysis was to determine what XGBoost learned about genomic features and how it used them. The model interpretation approach we used is based on SHAP values (Lundberg and Lee 2017; Lundberg *et al.* 2018; see *Methods*). SHAP values explain why the predicted response probability for one particular test example—1 TF-gene pair—differs from a default prediction, which is the probability that a randomly selected gene will be responsive to the perturbation of that TF. If the SHAP of some feature of a TF-gene pair is positive that feature increases the predicted probability that the gene will respond to perturbation; if negative, that feature decreases the predicted probability of response. The absolute value of the SHAP is an indicator of how much the feature influenced the predicted probability of response. Note that, even if the predicted probability of response is high, some features may still have negative SHAPs, indicating that their negative influence was outweighed by the positive influences of other features. Since there is a SHAP value for every feature of every instance, the dimensions of the SHAP matrix are the same as the dimensions of the feature matrix. To make generalizations about features, SHAPs or their absolute values can be summed over all instances or any subset of instances. All SHAP values reported here are on TF-gene pairs that serve as test examples in cross-validation, so their true labels have not been seen by the model.

**Fig. 2.**
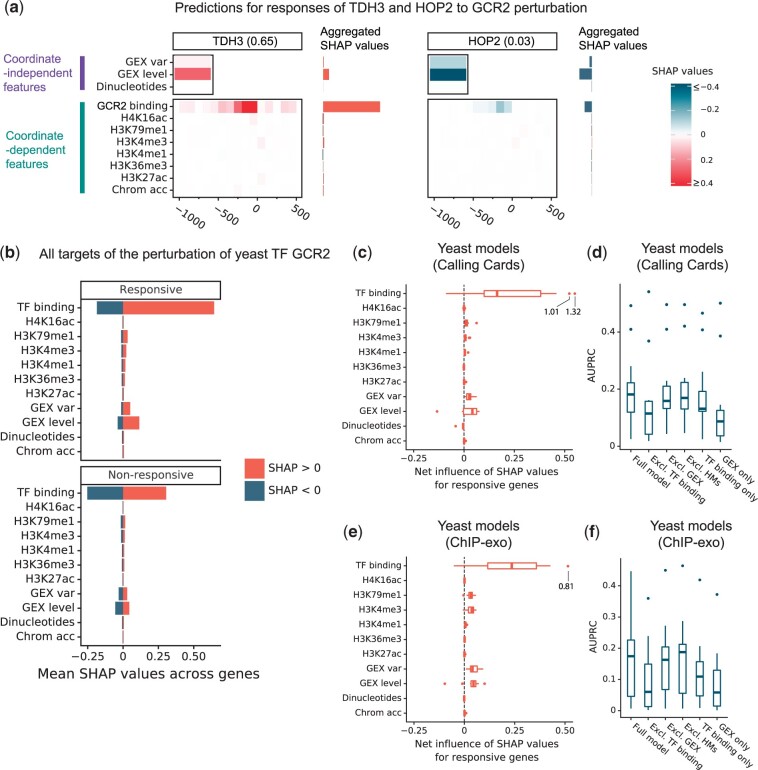
Quantification of yeast feature influences. a) An example of decomposing the predicted score using SHAP values. *TDH3* is a responsive target of yeast TF Gcr2 with predicted response probability of 0.65; *HOP2* is an unresponsive gene with predicted response probability of 0.03. The top panel shows the features that are independent of genomic coordinates; the bottom panel shows the features that depend on genomic coordinates. The right horizontal bars show the sums of SHAP values that are positive (red) or negative (blue), regardless of their genomic coordinates. b) Top: For yeast TF Gcr2, the positive (red) or negative (blue) SHAP values for each feature, summed over genomic positions relative to each gene and averaged over genes that respond to Gcr2 perturbation. Bottom: The same analysis for genes that do not respond to Gcr2 perturbation. c) Distribution across TFs of the “net influence” of each feature on predictions, averaged over responsive targets, when using Calling Cards data for TF binding. Net influence is the sum of all SHAP values for a feature, regardless of sign or genomic position. d) Accuracy comparison of yeast models trained on 5 sets of features: the model described previously (*Full model*), the model trained without TF binding features, the model without gene expression features, and the model without HMs, and the model trained only on gene expression features (*GEX only*). e) Same as (c) except for ChIP-exo data. f) Same as (d) except for ChIP-exo data.

Taking yeast TF Gcr2 as an example, Fig. 2a illustrated the SHAP values calculated for each feature of *TDH1*, a responsive gene, and *HOP2*, a nonresponsive gene. The primary factors that caused the model to predict that *TDH1* had a high probability of being responsive (0.65) are (1) the Gcr2 binding signal in the 500 bp upstream of the TSS and (2) *TDH1*’s preperturbation expression level (Fig. 2a, left, red). For *HOP2*, these positive influences were absent, so the predicted probability of response (0.03) is much closer to the expected probability of response for a random gene (∼0.07; see Fig. 2a, right). *HOP2*’s predicted probability of response is lower than 0.07 because its preperturbation expression level and its lack of Gcr2 binding signal. To aggregate these influences across the promoter regions of *TDH1* and *HOP2*, we separately summed all positive SHAP values for each feature, which are plotted in red to the right of the heatmaps, and all negative SHAP values for each feature, which are plotted in blue.

To get a sense of how feature values affected the model’s predictions for all genes in response to perturbation of a single TF, we first divided genes into responsive and nonresponsive. Within each group, for each feature, we separately summed its positive SHAP values from all promoter regions of all genes and its negative SHAP values (Fig. 2b). For Gcr2-responsive genes (Fig. 2b, top), Gcr2 binding data in the gene’s promoter tends to have a much bigger effect on predictions when it pushes the predicted probability of response up (red bar) than when it pushes the predicted probability of response down (blue bar). Comparing the red and blue bars for other features reveals net positive effects from preperturbation gene expression level and variation. HMs H3K79me1 and H3K4me3 have smaller positive influences. For genes that do not respond to the Gcr2 perturbation, the net influences of all features are close to zero (Fig. 2b, bottom), indicating that they do not push predictions for nonresponsive genes very far from the overall fraction of genes labeled responsive in the training data—7%. Specifically, the roughly equal red and blue bars in the bottom panel of Fig. 2b mean that, for nonresponsive genes, the TF binding features can increase or decrease the predicted probability that they will be responsive. They decrease it when the feature values indicate little or no evidence of binding and increase it when the feature values indicate stronger evidence of binding. The latter is fairly common because there are many bound genes that are not responsive [see Kang *et al.* (2020) and sources cited therein for a thorough investigation of that phenomenon].

To generalize from Gcr2 to all TFs, we calculated the net influences of features on predictions for genes that respond to perturbation of each TF and plotted the distributions across TFs (Fig. 2, c and e). This showed that the findings for Gcr2 generalize well to the other TFs. The biggest net influence was the binding signal from the perturbed TF, followed by gene expression level, gene expression variation, and HMs H3K79me1 and H3K4me3. Supplementary Fig. 8 shows the positive and negative influences of each feature on both responsive and nonresponsive genes. Complementary analysis of the effects of dropping feature classes from the model confirmed that TF binding features contribute most to the accuracy of the full model (Fig. 2, d and f). Note that TF binding location is the only feature that differentiates responses to different TFs, so the models that do not include binding make the same predictions for all TFs. Dropping gene expression features or HMs, on the other hand, had a very small effect. Models based on TF binding only (dropping all other features) have a modestly lower median AUPRC compared to the full model.

### In human cells, ChIP-seq peaks and epigenetic marks have little value for response prediction

**Fig. 3.**
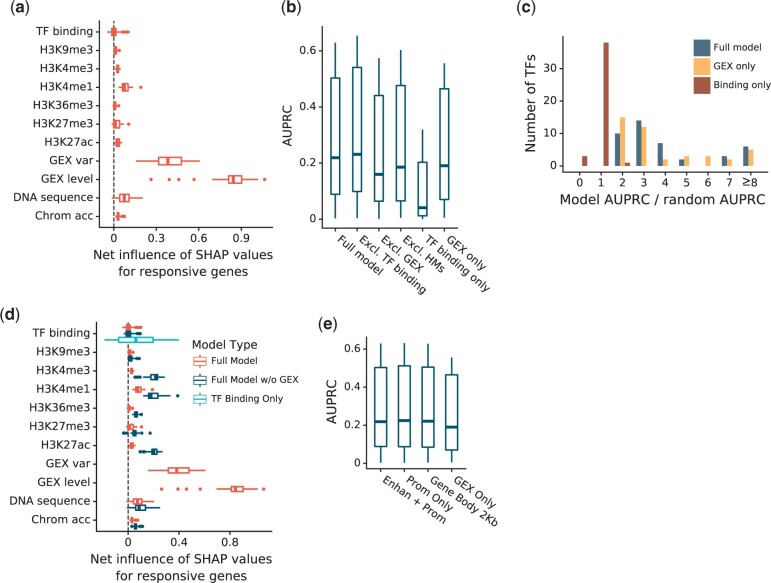
Quantification of human K562 feature influences. a) Distribution across K562 TFs of the net influence of each feature on predictions, averaged over responsive targets. b) Comparison of K562 model accuracy trained on 6 sets of input features: the complete set described previously (*Full model*), the complete set without TF binding features, the complete set without gene expression features, the complete set without HMs, only the TF binding features, and only the gene expression features (*GEX only*). c) Ratio of predictive accuracy to random expectation for the full model, the GEX only model, and binding only model. d) Net influences of features on predictions for responsive TFs in the full model, the GEX-only model, and the binding-only model. e) Comparison of model accuracy using 4 subsets of input features: the complete set (*Full model*), the complete set excluding enhancer features (*Prom only*), the complete set excluding enhancer and promoter upstream of the TSS (*Gene body 2Kb*), and gene expression features alone (*GEX only*).

Next, we summarized SHAP values for each human TF, focusing first on genes that respond to perturbation of the TF. Strikingly, ChIP-seq peaks for a TF, which reflect its binding locations, had essentially no net influence on predictions for genes that are in fact responsive (Fig. 3a). This is consistent with earlier studies (Gitter *et al.* 2009; Lenstra and Holstege 2012; Cusanovich *et al.* 2014; Kang *et al.* 2020). Gene expression level in unperturbed control samples was the most influential factor, followed by expression variation in the control samples. H3K4me1 and dinucleotide frequencies in the cis-regulatory DNA had very small influences on the predictions, while the effects of the other HMs and of chromatin accessibility were negligible. Analysis of nonresponsive genes yielded similar conclusions in all 3 human cell lines (Supplementary Fig. 8, c–e). Analysis of predictive accuracy also supported these conclusions: A model trained without the TF binding locations performed as well as one with those features, whereas a model using binding locations alone performed dramatically worse (Fig. 3b, Supplementary Files 3–5). Omitting the gene expression features, on the other hand, greatly reduced accuracy. In fact, a model using only the gene expression features was almost as accurate as the full model. This is also seen when comparing the AUPRC for each TF to the random expectation for the same TF (Fig. 3c, Supplementary Fig. 11, Supplementary Files 3–5). A model based on ChIP-Seq alone performed dramatically worse than either the full model or the expression-only model. With binding data alone, there was no TF for which actual AUPRC exceeded 2 times random, and for a few TFs, it was no better than random. This shows that the models can predict which genes will respond to perturbations of TFs with accuracy much better than chance using only features of the gene itself, primarily its expression, without any information about which TF was perturbed.

We hypothesized that HMs may lack influence in the model because the gene expression features summarize any useful information provided by HMs, as well as other aspects of a gene’s epigenetic state. To test this, we trained a model without the gene expression features and analyzed the influence of the remaining features on predictions for genes that are in fact responsive (Fig. 3d, Supplementary Fig. 10). Removing the gene expression features from the model did increase the influence of H3K27ac, H3K4me3, and H3K4me1, supporting our hypothesis. However, the model without gene expression features has low accuracy (median AUPRC 0.11), so the predictive value of HMs is small.

These findings drove us to investigate the utility of features mapped to various regions of the cis-regulatory DNA associated with each gene. When we dropped the TF binding signal, histone modifications, dinucleotide frequencies, and chromatin accessibility from the enhancer regions associated with each gene, the effect on prediction accuracy was negligible (mean AUPRC *increased* by 0.005, Fig. 3e). We then tried dropping all features from both the enhancers and the promoter regions upstream of the TSS, leaving only the first 2 kb of the gene body. Again, the effect on accuracy was negligible (mean *AUPRC increased* by 0.001 relative to the full model). Dropping these genomic features entirely, leaving only GEX features, also had little effect (mean AUPRC decreased by 0.033, or 12% of the full model’s mean AUPRC). While the TF binding signal and epigenetic features significantly enhanced prediction accuracy in yeast, they had little predictive value in the human data. What predictive value they did have was entirely due to features mapped to the 2 kb downstream of the TSS.

### In yeast cells, TF binding locations and strengths discriminate between bound genes that are responsive and those that are not

**Fig. 4.**
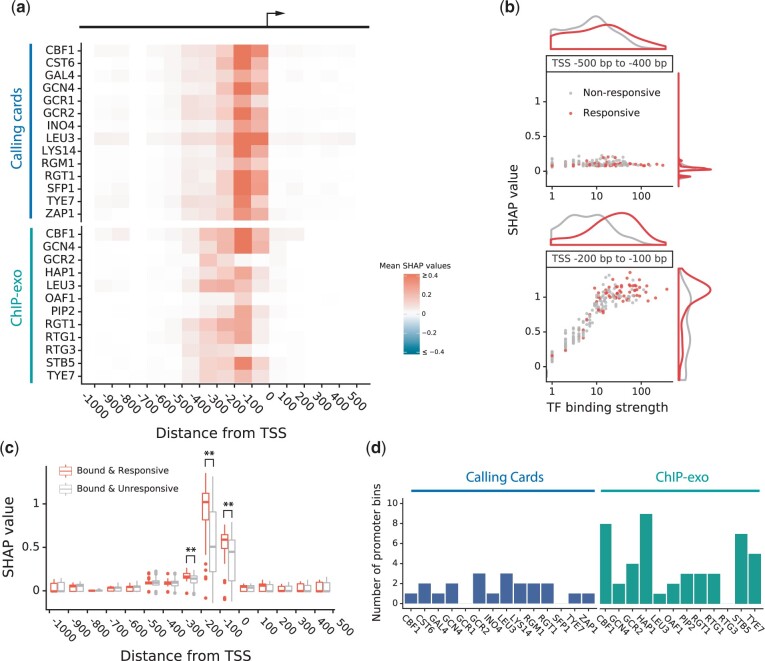
TF binding features in yeast models. a) Heatmap of the influence of yeast TF binding signals along regulatory DNA. Each pixel is the mean signed SHAP value over all target genes that were bound by the perturbed TFs. b) Comparison of 2 upstream bins [(−500, −400) and (−200, −100)] of TF Leu3. Among the genes that are bound by Leu3, the responsive genes are more clearly distinguished from the unresponsive ones in the (−200, −100) bin. This shows that even within 500 bp of the TSS, Leu3 binding near the TSS is more likely to be functional than Leu3 binding further away. c) Comparison of feature influences on responsive and unresponsive targets that were bound by Leu3. *P* < 0.01 (*), *P < *0.001(**), Wilcoxon rank-sum test. The significant differences show that responsive genes are bound more strongly than unresponsive genes, even among significantly bound genes. Furthermore, all significant effects of binding strength are within 300 bp upstream of the TSS. d) Number of 100 bp promoter bins in which the bound and responsive genes have significantly higher SHAP values for TF binding (*P < *0.05) than the bound but nonresponsive genes.

The most common use of in vivo binding location data is to classify genes into those whose regulatory DNA is or is not bound by the TF. This typically yields a large set of genes that are bound by the TF at a statistically significant level but are not responsive to perturbation of that TF (Gitter *et al.* 2009; Lenstra and Holstege 2012; Cusanovich *et al.* 2014; Kang *et al.* 2020). Thus, we investigated whether the model could use the strength and location of the binding signal to better predict which bound genes would be responsive. In Fig. 4a, each row shows SHAP values of the TF binding signal in each promoter bin, averaged across the genes that were significantly bound by the perturbed TF (see *Methods*). For all TFs, the binding signal in the 500 bp upstream of a gene’s TSS influenced the model toward predicting (correctly) that the gene would respond to the perturbation. Strikingly, the positive influence of binding was strongest in the region between 100 and 200 bp upstream of the TSS and dropped off rapidly with increasing distance. Using calling cards data for TF Leu3 as a typical example, stronger binding signals were more influential than weaker ones and signals of the same strength were more influential in the region 100–200 bp upstream of the TSS than in the region 400–500 bp upstream (Fig. 4b). SHAP values in the closest 3 promoter bins were significantly higher among the bound and responsive group than in the bound but unresponsive group (Fig. 4c). For most TFs, a similar pattern was found in one or more promoter bins (Fig. 4d, Supplementary Fig. 9). Thus, the strength and location of the binding signal are meaningful predictors of whether significantly bound genes will respond to the perturbation, consistent with our earlier findings (Kang *et al.* 2020).

### Highly expressed genes and genes with high expression variation are more likely to be responsive

**Fig. 5.**
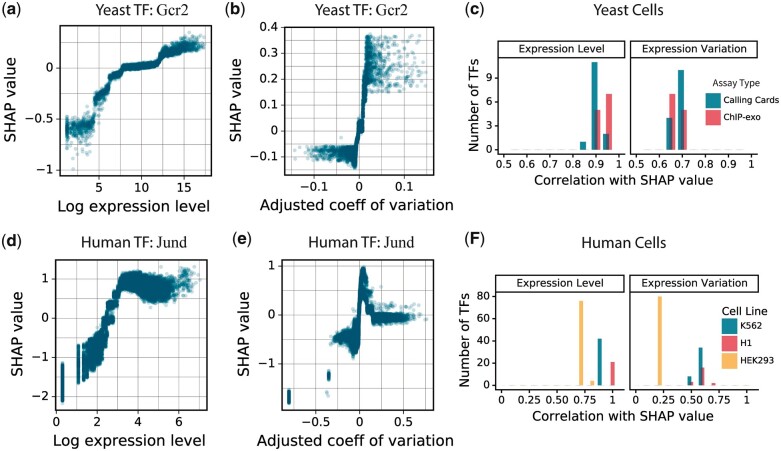
Gene expression features. a) Relationship between feature input and SHAP values of gene expression level for perturbation of yeast TF Gcr2. The model predicts that more highly expressed genes are more likely to be responsive. b) Relationship between feature input and SHAP values of gene expression variation for the same model as in (a). The model predicts that genes whose expression levels are more variable, after correction for their average expression level, are more likely to be responsive to Gcr2 perturbation. c) The distribution of the correlations between input and SHAP values for the 2 expression-related features in yeast cells when grouped by TF binding assay type. For all TFs, both expression level and expression variation are positively correlated with response to a perturbation. On average, expression level is more positively correlated than expression variation. All correlations are statistically significant. d) Same as (a) for predicted responses to TF Jund in K562 cells. e) Same as (b) for predicted responses to TF Jund in K562 cells. f) Same as (c) for human K562, H1 cells, and HEK293 cells. All correlations are statistically significant.

Given the predictive power of gene expression level and variation, we investigated how the model used these features. Starting with yeast TF Gcr2, we noted a monotonic relationship in which the more highly a gene was expressed before the perturbation, the more the model expected it to respond to a perturbation (Fig. 5a). We also noted that the more a gene’s expression varied from one preperturbation sample to another, the more the model expected it to respond (Fig. 5b). This was not due to the relationship between expression level and expression variation, which we removed by fitting a model that predicts expression variation from expression level and using the residuals from that model as our variation feature (Supplementary Fig. 2). For all yeast TFs, both expression level and expression variation are positively correlated with SHAP value—higher expression level and expression variation push the model to predict a higher probability of response (Fig. 5c). The same pattern holds for human TFs (Fig. 5, d–f).

### HMs downstream of the TSS are more predictive of responsiveness than upstream HMs

**Fig. 6.**
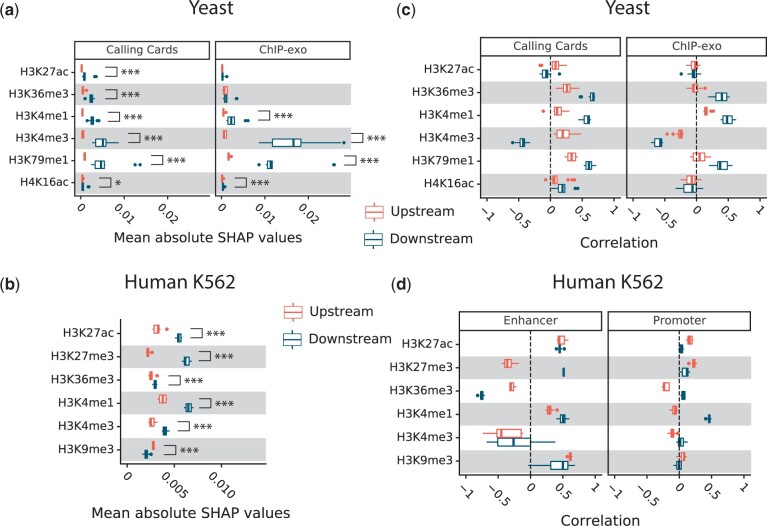
Epigenetic features. a) Comparison of the global importance of HM features in the regions upstream or downstream of the TSS in yeast. The distribution across TFs is shown. For each TF, the global importance of each feature is the absolute SHAP value for that feature averaged across all genes and all promoter bins upstream or downstream of the TSS. *P* < 0.05 (*), *P < *0.001(***), Wilcoxon rank-sum test. b) Same as (a) but for K562 cells. c) Correlation of yeast histone modification signals and their corresponding SHAP values averaged over upstream or downstream bins. d) Same as (c) but for K562 cells.

We showed above that for human TFs, models trained using coordinate-dependent features in enhancers, the 2 kb upstream of the 5′-end TSS, and the 2 kb downstream of the 5′-end TSS were no more accurate than those that used only the downstream features (Fig. 3e). For both yeast and human, the downstream HMs had a much greater influence on the predictions than the upstream marks (Fig. 6, a and b). This was quantified for each TF by the mean absolute SHAP values across all genes. Among the 6 HMs, we analyzed in yeast, downstream H3K79me1 and downstream H3K4me3 had the biggest influence on predictions, followed by downstream H3K4me1. When combined with calling cards data, all HMs were significantly more important when they occur downstream of the TSS compared to upstream of the TSS (Fig. 6a). For most HMs, a stronger signal increased the predicted probability of response, but downstream H3K4me3 reduced the probability of response (Fig. 6c). For K562 cells we did not have data on H3K79me1. Downstream H3K4me3 and H3K4me1 are the 2 most influential marks, followed by downstream H3K27ac (Fig. 6b). As with yeast, downstream H3K4me3 decreased the probability of response while downstream H3K4me1 increased probability of response (Fig. 6d). However, it is important to note that the influence of HMs in both models was very small, affecting the predicted probability of response by no more than 0.02 (Fig. 6, a and b, horizontal axes).

## Discussion

Determining which genes are regulated by each TF in an organism is a fundamental goal of regulatory systems biology. Furthermore, the ability to predict which genes will respond to perturbation of a TF serves as a benchmark for how well we understand the TF network. There is a body of work focused on predicting gene expression level (Middendorf *et al.* 2004; Ouyang *et al.* 2009; Karlić *et al.* 2010; Cheng *et al.* 2011; Dong *et al.* 2012; McLeay *et al.* 2012; Zhou *et al.* 2014; González *et al.* 2015; Singh *et al.* 2016; Schmidt *et al.* 2017; Kelley *et al.* 2018; Zhou *et al.* 2018; Crow *et al.* 2019; Read *et al.* 2019; Washburn *et al.* 2019; Agarwal and Shendure 2020; Sigalova *et al.* 2020; Tasaki *et al.* 2020), but this is a very different task from predicting the response of expression level to TF perturbations by using only data from unperturbed cells.

Data on where in the genome each TF binds were expected to be of great value in determining its targets, but multiple studies have shown that in the available large ChIP-chip and ChIP-seq datasets, the genes whose regulatory DNA a TF binds do not correspond well to those that respond to perturbation of the TF (Gitter *et al.* 2009; Lenstra and Holstege 2012; Cusanovich *et al.* 2014; Kang *et al.* 2020). We followed up on these observations by training machine learning models to predict which genes would respond to perturbation of a TF, given data on the TF’s binding locations and several features reflecting the gene’s epigenetic context. We found that data on yeast TF binding locations obtained by the calling cards method (Wang *et al.* 2011; Shively *et al.* 2019; Kang *et al.* 2020) and the ChIP-exo method (Bergenholm *et al.* 2018; Holland *et al.* 2019) are useful for predicting which genes will respond to a perturbation of the TF. In fact, the binding location was the most influential and valuable among the features we provided (Fig. 2). Since earlier ChIP-chip data on yeast are known to correspond poorly to perturbation response, we conclude that the newer technologies are yielding better results. Binding signals influenced predictions mainly in the 500 bp upstream of the TSS, suggesting that this is the extent of functional yeast promoter regions (Fig. 4). Even among genes with significant binding signal for a TF in their promoter, the strength and location of the signal helped to differentiate between functional and nonfunctional binding (Fig 3, c and d). In data on human cells, however, the situation was strikingly different. The models did not identify patterns in ChIP-seq data on the perturbed TF that were useful for predicting which genes would respond to the perturbation (Fig. 3, Supplementary Files 3–5).

We also investigated the predictive value of selected HMs and chromatin accessibility features. In yeast, these features had very little predictive value (Fig. 2). In human, they also had little value in models that included preperturbation gene expression level and variation (Figs. 3, a and b and 6, c and d). HM features had a larger (though still small) impact in models that did not include gene expression (GEX) features (Fig. 3d and Supplementary Fig. 10, discussed below). HMs were most influential when they occurred downstream of the TSS, in the gene body. In fact, dropping all features that mapped to the enhancers and the promoter region upstream of the TSS had only a small impact on predictive accuracy in K562 cells (Fig. 3e). This surprising observation likely reflects both the low utility of the existing ChIP-seq data and incomplete knowledge of enhancer locations and enhancer–gene associations. Future datasets on TF binding locations and enhancer–gene associations will likely reveal at least some predictive power for enhancer features.

In human data, preperturbation gene expression level was by far the best predictor of which genes would respond to a perturbation, followed by expression variation. Although it has been previously reported that expression variation predicts responsiveness to perturbations in general (Sigalova *et al.* 2020), the dominance of expression level as a predictor surprised us. In fact, a model using only these 2 features predicted responses in human cells almost as well as the full model, which includes TF ChIP-seq, HMs, chromatin accessibility, and dinucleotide frequencies (Fig. 3, b and c, Supplementary Files 3–5). Genes that were expressed at a higher level and genes that showed more variability in their expression level were more likely to respond to perturbations (Fig. 5). We hypothesize that these features are readouts of molecular features of each gene’s sequence context and/or epigenetic state that have limited predictive power individually, but much greater predictive power when aggregated by their effects on gene expression level and variation. This hypothesis is supported by the observation that the influence of several HMs increases when GEX features are omitted from the model, though these influences are still small compared to the impact of GEX features (Fig. 3d, Supplementary Fig. 10). However, the epigenetic state that is reflected in the GEX features is not as simple as open chromatin vs closed chromatin, since chromatin accessibility has little influence even in the absence of GEX features. Previous studies suggest that several sequence features, including the presence of a TATA box in the promoter (Blake *et al.* 2006; Ravarani *et al.* 2016; Sigalova *et al.* 2020) and the GC content of the promoter (Morgan and Marioni 2018; Sigalova *et al.* 2020) influence expression variation. Identifying additional sequence elements or epigenetic states that are reflected in GEX features and showing that they can predict perturbation response is an important direction for future research.

In human data, the predictive power of features that are independent of which TF is perturbed—GEX features, HMs, chromatin accessibility, and dinucleotide sequence—shows that some genes are poised to respond to perturbations while others are not (Fig. 3, a and b, Supplementary Fig. 10), consistent with the findings of Crow *et al.* (2019). If you want to predict which genes will respond to perturbation of a human TF, it is not enough to know where the TF binds—you must also know whether the gene is predisposed to respond to perturbations. However, the cell type- and perturbation-independent “differential expression prior” developed by Crow *et al.* is not a good predictor of which genes will respond to perturbation of a TF in our dataset (Supplementary Fig. 12). An important direction for future research is to discover the extent to which a gene’s predisposition to respond to TF perturbations is an epigenetic feature that depends on cell type and conditions or an inherent feature of the gene.

For most TFs, the AUPRC for the trained models was multiple times the random expectation (Fig. 3c, Supplementary Fig. 11), but it was still small compared to anything that could be considered an accurate predictive model (Figs. 2, d and f and 3, b and e; Supplementary Fig. 3). Neither the yeast nor the human model can predict which genes will respond to a TF perturbation with high accuracy, even when provided with the binding locations of the perturbed TF and a host of epigenetic features. This general observation remains true for datasets associated with 3 human cell lines, despite some differences in accuracy (compare Fig. 1d to Supplementary Fig. 5) and many differences among the perturbation and binding datasets used (Table 1). For the human data, it is possible that performance would be better if the gene expression were measured shortly after inducing a perturbation, instead of the longer intervals used in the current datasets (see Table 1). One possible route to improving accuracy would be to provide binding location features for other TFs. For example, some genes may respond to perturbation of TF that does not bind in their regulatory DNA because the perturbed TF regulates another TF that does bind in their regulatory DNA. In principle, a learning algorithm given the binding locations of all TFs might be able to discover such network effects itself, but in practice, the number of features is likely to be too large compared to the number of training examples for responses to perturbation of a single TF. An alternative would be to map the TF network externally, using NetProphet 2, Inferelator, or other network inference algorithms (Huynh-Thu *et al.* 2010; Greenfield *et al.* 2013; Roy *et al.* 2013; Kang *et al.* 2018). The response prediction algorithm could then be provided with binding location data on TFs that the perturbed TF regulates. With those data, it could learn that genes respond to perturbation of a given TF if they are bound by another TF. Expanding to other types of TF–TF interactions, binding data might also be for TFs that interact physically with the perturbed TF, according to protein–protein interaction maps (Oughtred *et al.* 2019; Szklarczyk *et al.* 2019).

For human cells, the inability to predict responsiveness may also reflect limitations of the technologies used for measuring TF binding locations and perturbing TFs, as well as limited knowledge of enhancer locations and enhancer–gene associations. It may also be possible to improve on the way enhancer-associated features were coded, enabling models to better utilize HMs and chromatin accessibility for determining which enhancers are active in a given sample of cells (Fullwood and Ruan 2009; Lonsdale *et al.* 2013; Fulco *et al.* 2016; Mumbach *et al.* 2016; Aguet *et al.* 2017; Klann *et al.* 2017; Mumbach *et al.* 2017; Simeonov *et al.* 2017). Other types of data, such as levels of enhancer-associated transcription, may also help (Core *et al.* 2014; Mahat *et al.* 2016; Azofeifa *et al.* 2018; Tome *et al.* 2018). For yeast, however, these explanations are less applicable. Obtaining the right genomic data and developing the right models for predicting which yeast genes will respond to the perturbation of a TF remains a major challenge. Progress in overcoming this challenge will serve as a benchmark for our understanding of regulatory systems biology.

## Data availability

Code is available at https://github.com/BrentLab/TFPertRespExplainer. All data were downloaded from the publicly available sources indicated in *Methods*.

Supplemental material is available at *G3* online.

## Funding

This work was supported by award GM141012 from the National Institute of General Medical Sciences within the National Insitutes of Health.

## Conflicts of interest

None declared.

## Supplementary Material

jkac144_Supplemental_Figures

jkac144_Supplemental_File_S1

jkac144_Supplemental_File_S2

jkac144_Supplemental_File_S3

jkac144_Supplemental_File_S4

jkac144_Supplemental_File_S5

jkac144_Supplemental_Methods
