# Supplementary material for: Predicting which genes will respond to transcription factor perturbations
Source: G3 (Bethesda). 2022 Jun 6;12(8):jkac144. doi: 10.1093/g3journal/jkac144 (PMC9339286; doi:10.1093/g3journal/jkac144)
Supplement: jkac144_Supplemental_Figures [file jkac144_supplemental_figures.pdf]

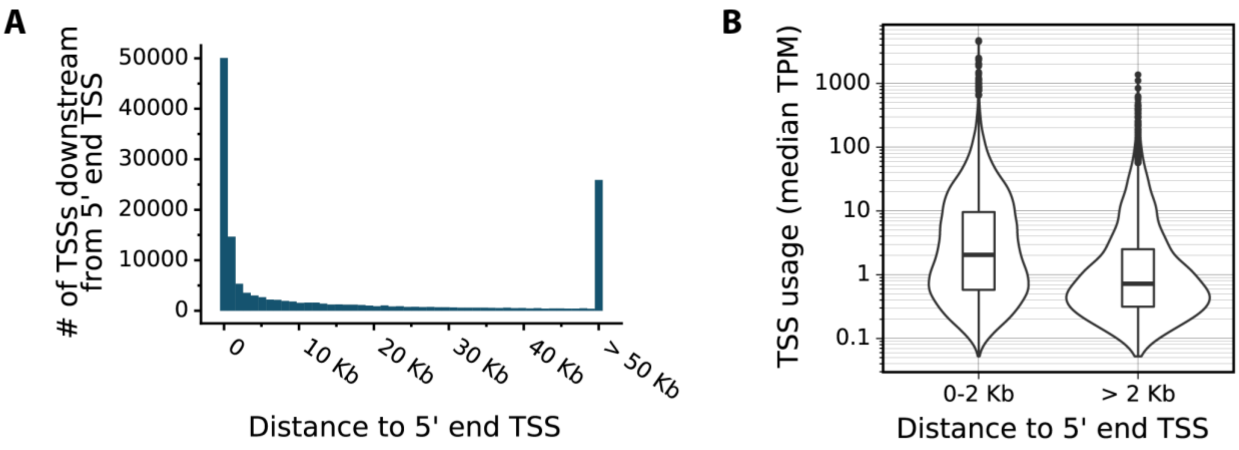

Figure S1. Statistics on human TSSs. (A) Distances between each 5’ end TSS and other downstream TSSs of the corresponding gene. Among the downstream TSSs for all genes, ~47% of them are within the 2 Kb range of the 5’ end TSS. (B) Relationship between TSS usage and distance on a logarithmic scale. TSS usage for each TSS is represented as the median Tags Per Million (TPM) level across all samples in Fantom5 CAGE expression data (Forrest et al. 2014; Lizio et al. 2019). The median TPM for the TSSs within 2 Kb range to their 5’ end TSS (including these 5’ end TSS themselves) is approximately three times of the median TPM for the TSSs that are more than 2 Kb away from their corresponding 5’ end TSS. This indicates that more distant annotated TSS’s, which we treated as enhancers, are typically transcribed at a much lower level than TSS’s included in the 4 Kb region around the 5’ TSS, which we treated as part of the promoter.

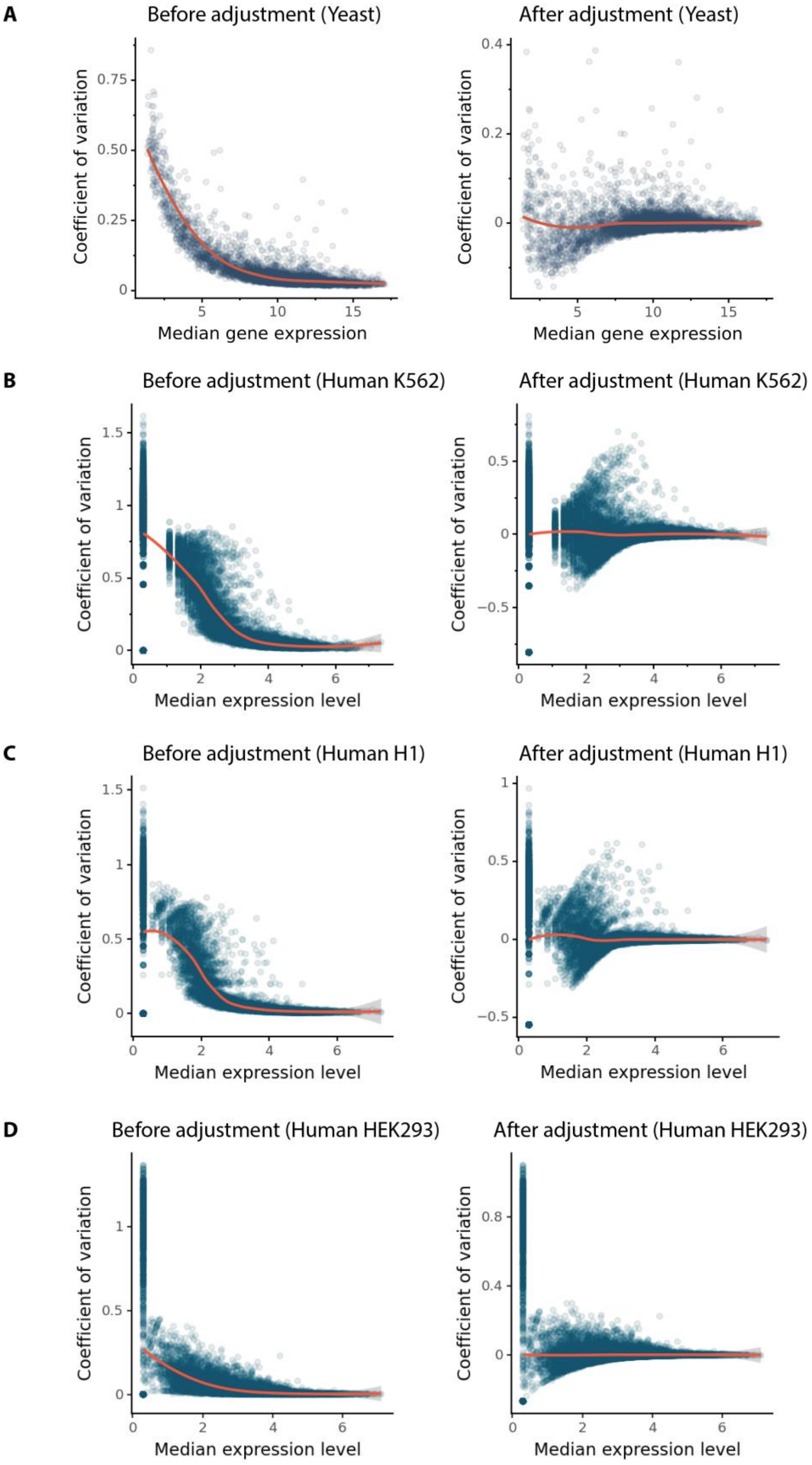
A
Before adjustment (Yeast)
After adjustment (Yeast)
Coefficient of variation
Median gene expression
B
Before adjustment (Human K562)
After adjustment (Human K562)
Coefficient of variation
Median expression level
C
Before adjustment (Human H1)
After adjustment (Human H1)
Coefficient of variation
Median expression level
D
Before adjustment (Human HEK293)
After adjustment (Human HEK293)
Coefficient of variation
Median expression level

Figure S2. (A) Expression variation for yeast cells. Left: Relationship between the median expression level of each gene across pre-perturbation (or control) conditions and its expression variation measured by the coefficient of variation. Orange curve was fitted using locally estimated scatterplot smoothing (LOESS) regression. Right: Expression variation adjusted for the median expression level by taking the residual of LOESS regression (the orange curve from left). (B) Expression variation for human K562 cells. Same analysis as in (A). (C) Expression variation for human H1 cells. Same analysis as in (A). (D) Expression variation for human HEK293 cells. Same analysis as in (A).

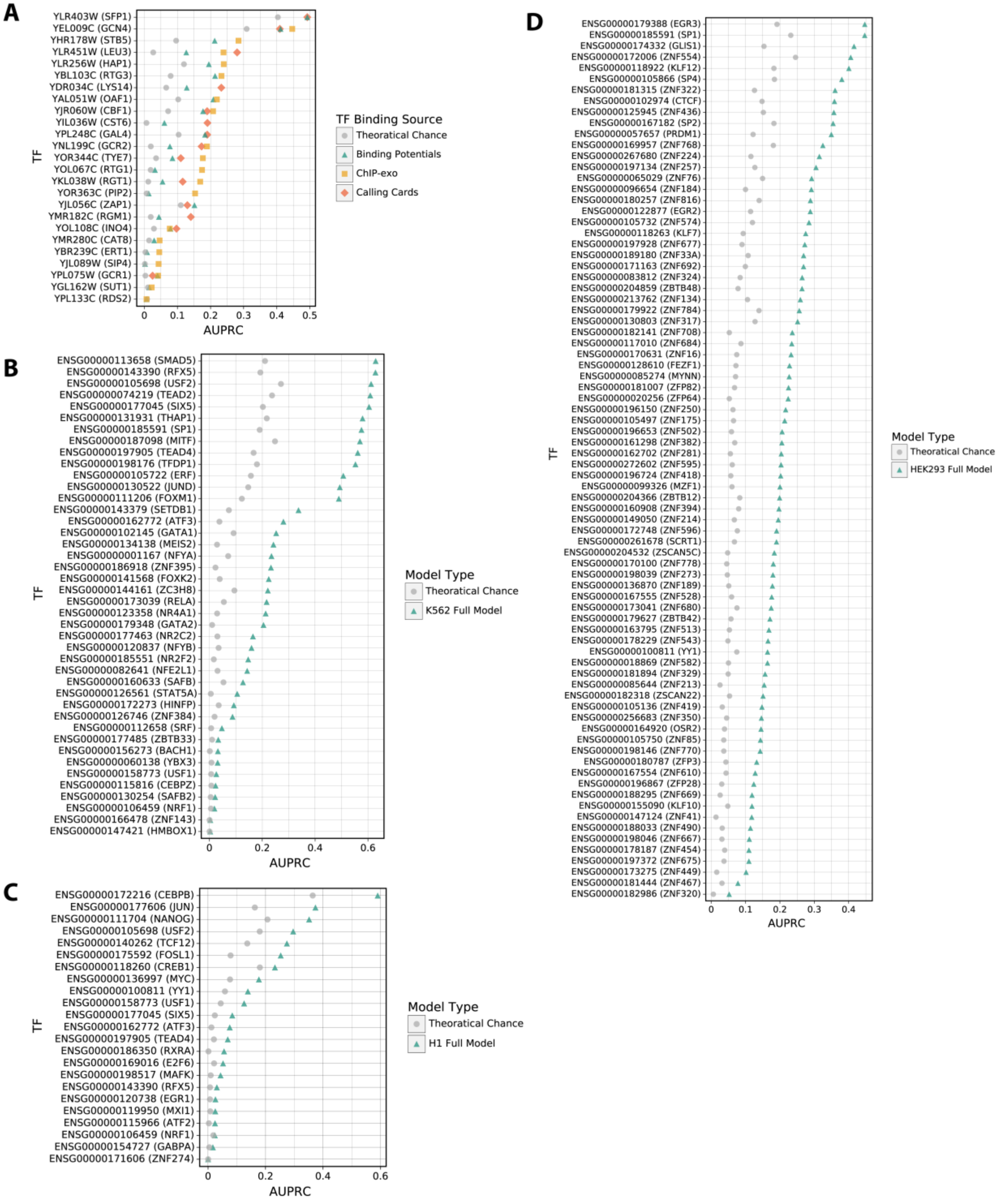

Figure S3. (A) Performance of individual yeast TF models that were trained on different types of TF binding data and binding potentials. (B) Performance of individual human K562 TF models. (C) Same as (B) except for H1 models. (D) Same as (B) except for HEK293 models.

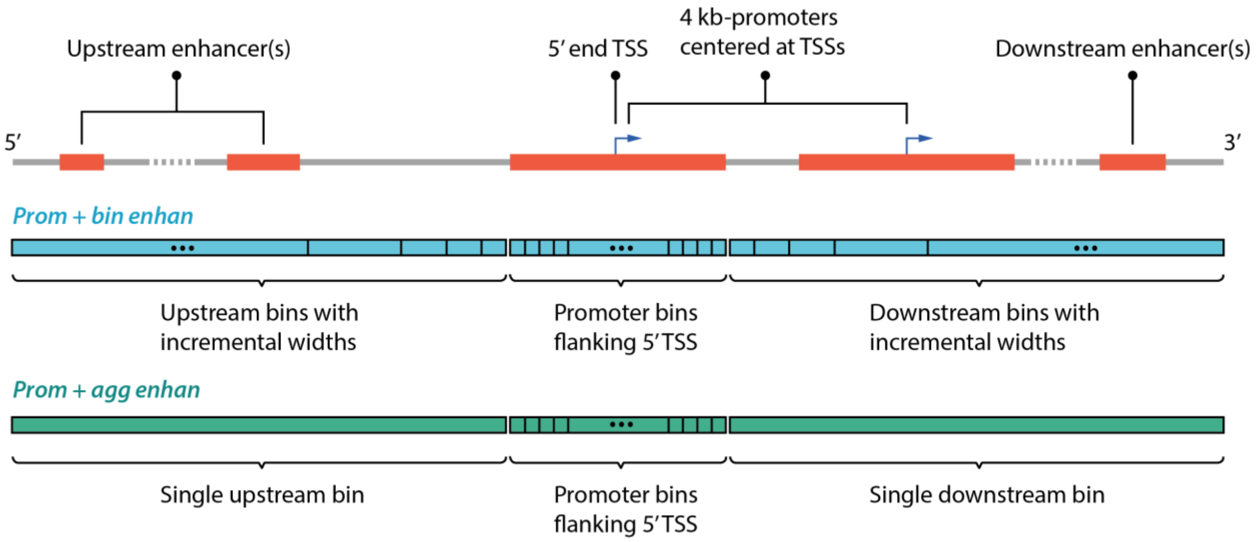

Figure S4. Definition of human cis-regulatory regions. The top panel illustrates a 1 Mb region centered at the 5' end TSS of a gene. Orange boxes indicate the enhancers linked to the gene, and the 4 Kb promoter(s) centered around the gene's TSS(s). The bottom two panels illustrate the approaches for binning the 1Mb cis-regulatory region. *Prom + bin enhan* (blue) includes 40 equal-sized bins of the promoter centered around the 5' TSS, and 45 bins with incremental widths for the upstream regions between -500 Kb and -2 Kb and another 45 bins for the downstream regions between 2 Kb and 500 Kb respectively. As the distance between the distal bin and TSS increases, the width of the bin increases exponentially. *Prom + agg enhan* (green) includes 40 equal-sized bins of the promoter centered around the 5' TSS, one single upstream bin covering the entire region between -500 Kb and -2 Kb, and one single downstream bin covering the region between 2 Kb and 500 Kb. The signals of each coordinate-dependent feature that are mapped to the defined cis-regulatory regions (orange, top panel) linked to the corresponding bins according to the genomic coordinates. Within each bin, the signals are summed into a single aggregated input value.

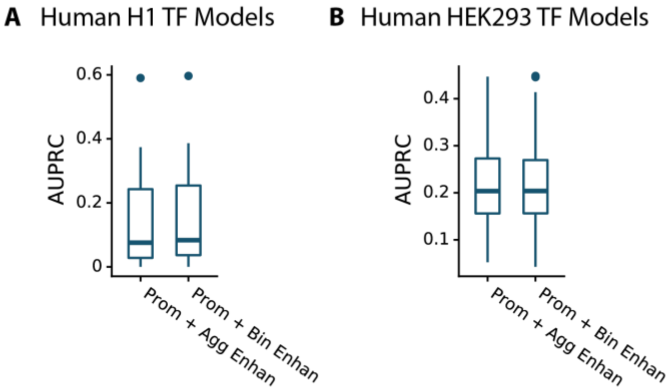

Figure S5. (A) Model accuracy on human H1 cells using two methods of aggregating data from enhancers associated with each gene. (B) Same as (A) except for human HEK293 cells.

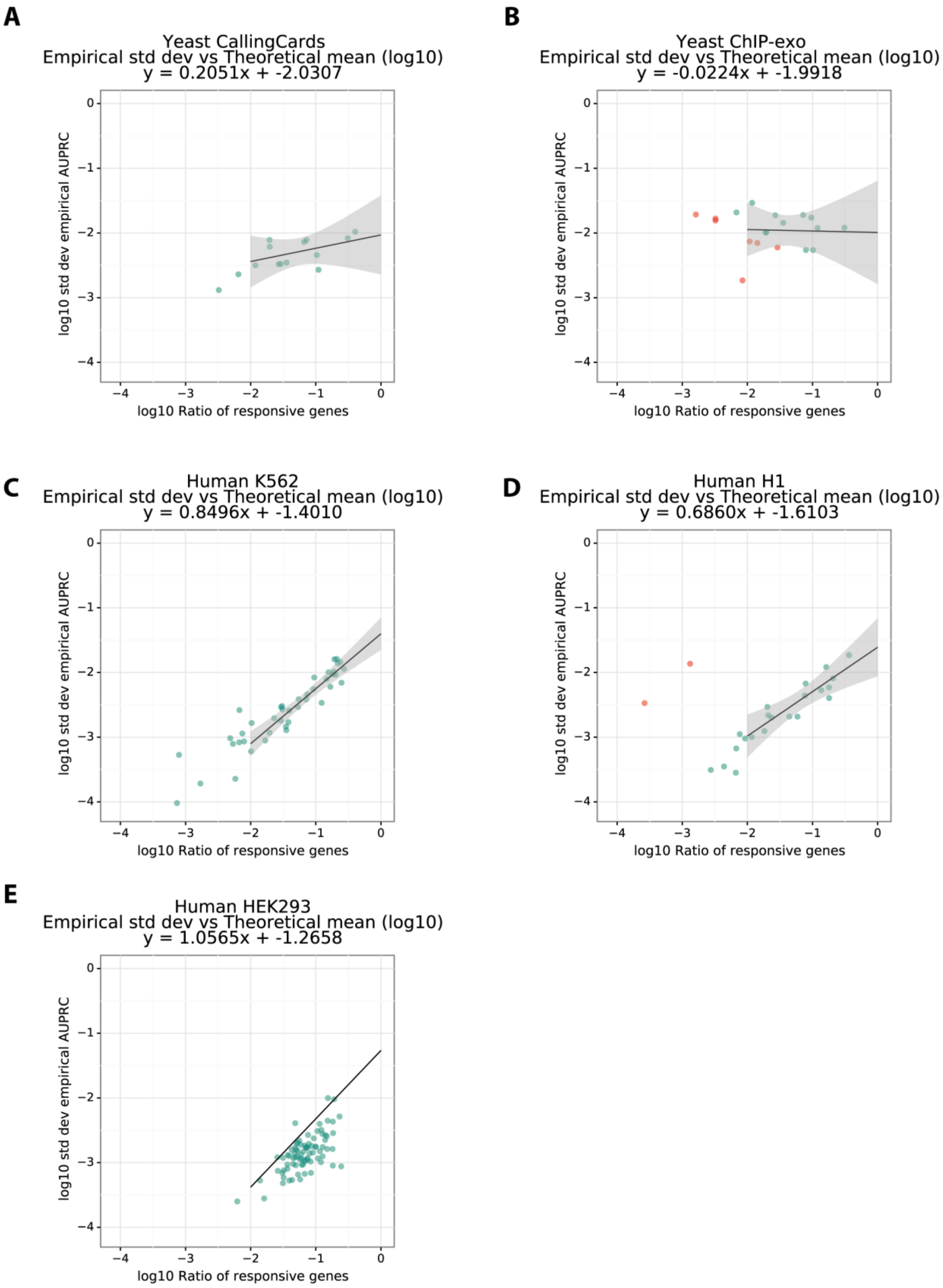
A
Yeast CallingCards
Empirical std dev vs Theoretical mean (log10)
y = 0.2051x + -2.0307
log10 std dev empirical AUPRC
log10 Ratio of responsive genes
B
Yeast ChIP-exo
Empirical std dev vs Theoretical mean (log10)
y = -0.0224x + -1.9918
log10 std dev empirical AUPRC
log10 Ratio of responsive genes
C
Human K562
Empirical std dev vs Theoretical mean (log10)
y = 0.8496x + -1.4010
log10 std dev empirical AUPRC
log10 Ratio of responsive genes
D
Human H1
Empirical std dev vs Theoretical mean (log10)
y = 0.6860x + -1.6103
log10 std dev empirical AUPRC
log10 Ratio of responsive genes
E
Human HEK293
Empirical std dev vs Theoretical mean (log10)
y = 1.0565x + -1.2658
log10 std dev empirical AUPRC
log10 Ratio of responsive genes

Figure S6. (A) Comparison of the standard deviations of empirical AUPRCs from the models trained on permuted data to the fraction of genes that responded to the perturbation for yeast Calling Cards data. The ratio of responsive genes is the expected value of the AUPRC for models trained on randomly permuted data. (B) Same as (A) except for yeast TF models trained on ChIP-exo. Red points represent 7 TFs with P>0.001 which were dropped from subsequent analyses. (C) Same as (A) except for human K562 TF models. (D) Same as (A) except for human H1 TF models. (E) Same as (A) except for human HEK293 TF models.

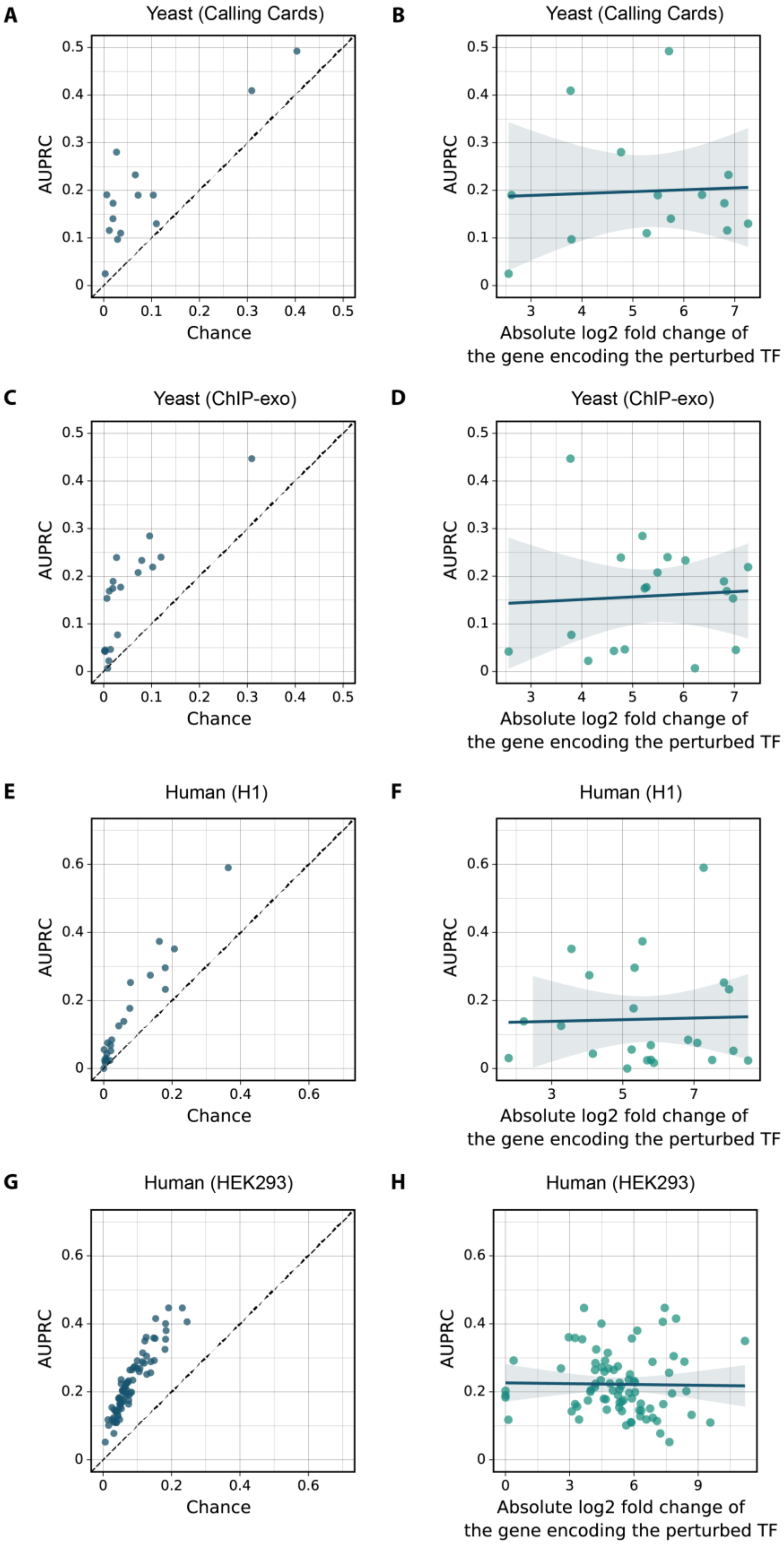
A
Yeast (Calling Cards)
AUPRC
Chance
B
Yeast (Calling Cards)
AUPRC
Absolute log2 fold change of the gene encoding the perturbed TF
C
Yeast (ChIP-exo)
AUPRC
Chance
D
Yeast (ChIP-exo)
AUPRC
Absolute log2 fold change of the gene encoding the perturbed TF
E
Human (H1)
AUPRC
Chance
F
Human (H1)
AUPRC
Absolute log2 fold change of the gene encoding the perturbed TF
G
Human (HEK293)
AUPRC
Chance
H
Human (HEK293)
AUPRC
Absolute log2 fold change of the gene encoding the perturbed TF

Figure S7. (A) Comparison of model accuracy on each TF and theoretical chance for yeast TF models trained on Calling Cards data. (B) Comparison of model accuracy on each yeast TF and the efficacy of TF perturbation defined by the absolute log2 fold change of the gene encoding the perturbed TF. (C) Same as (A) except for yeast TF models trained on ChIP-exo data. (D) Same as (B) except for yeast TF models trained on ChIP-exo data. (E) Same as (A) except for human H1 TF models. (F) Same as (B) except for H1 TF models. (G) Same as (A) except for human HEK293 TF models. (H) Same as (B) except for HEK293 TF models.

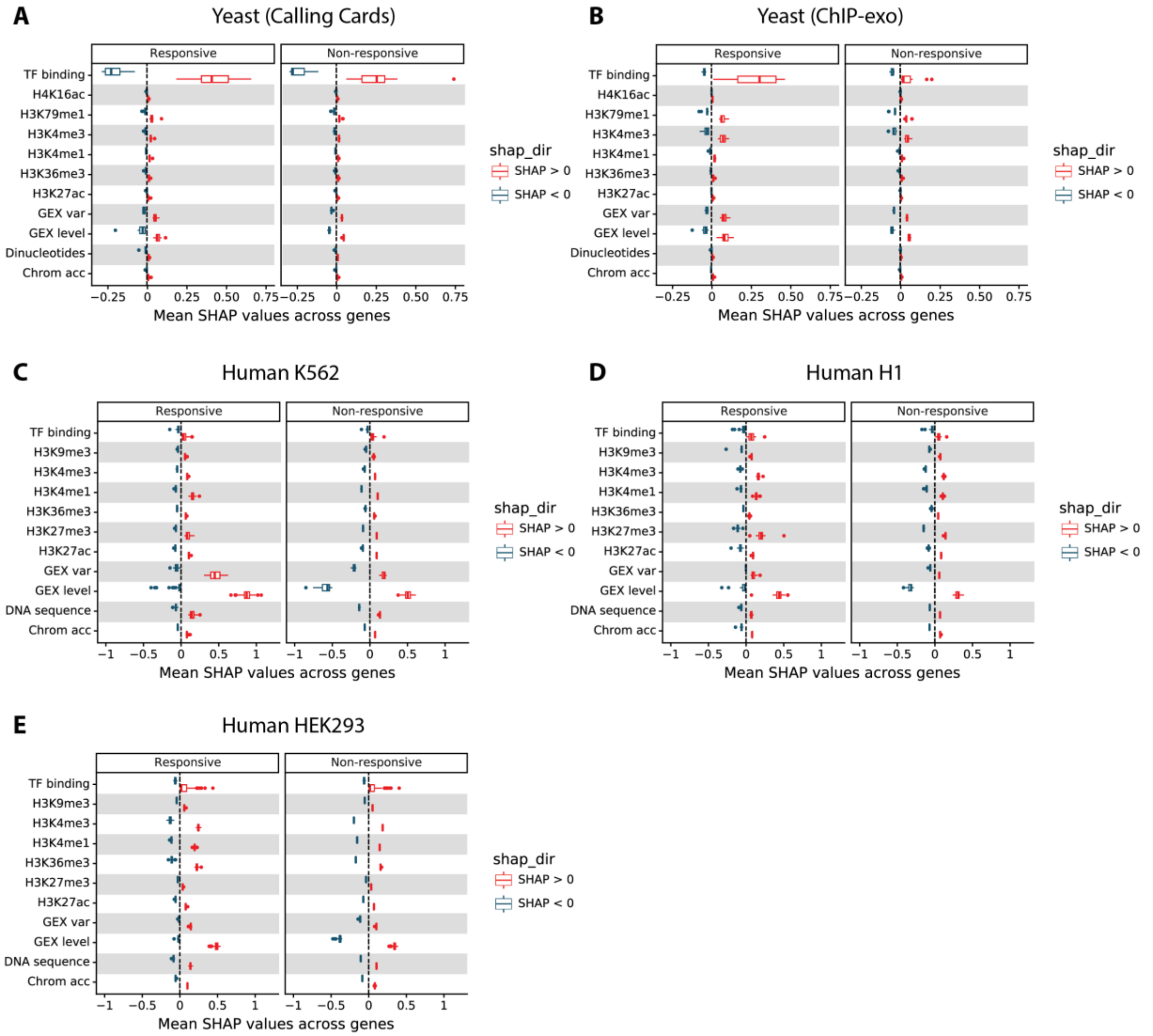

Figure S8. (A) Mean SHAP values for all genes that are responsive or unresponsive to perturbation of a TF, using yeast Calling Cards data. Box and whiskers indicate the distribution of TFs. (B) Same as (A) for yeast ChIP-exo data. (C) Same as (A) for human K562 data. (D) Same as (A) for H1 data. (E) Same as (A) for HEK293 data.

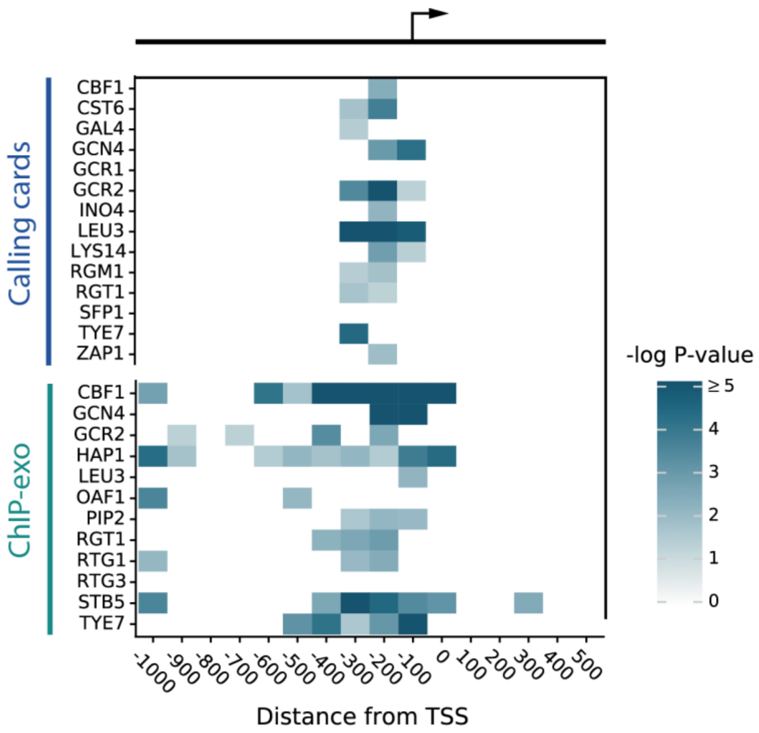

Figure S9. Heatmap of the statistics that indicate the degree to which the bound but unresponsive genes have insufficient TF occupancy. The P-values for all bins in each row (TF) were estimated using the method in Figure 4C. Bins with P-values greater than or equal to 0.05 are white.

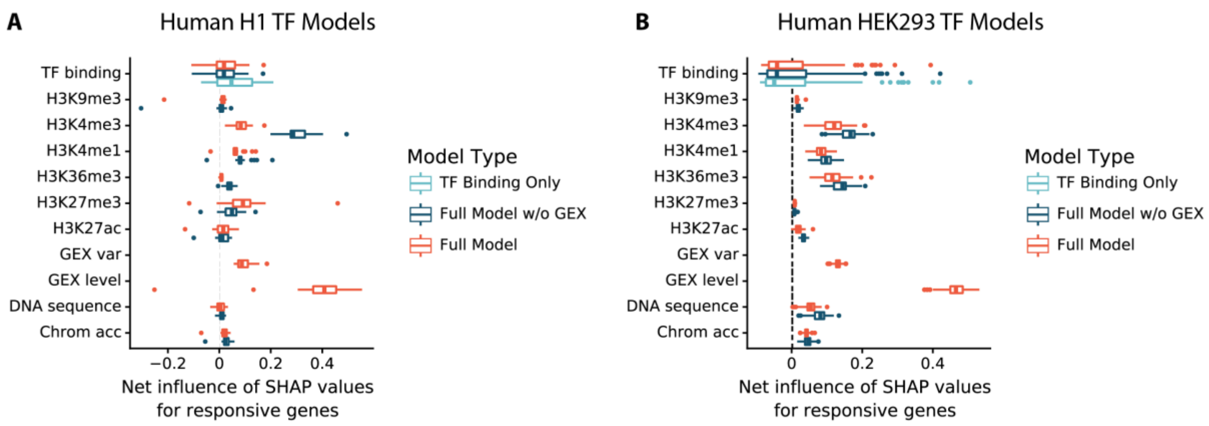

Figure S10. (A) Comparison of net influences of features on predictions for responsive human H1 genes. Models were trained with gene expression features (*Full model*) or without. (B) Same as (A) except for human HEK293 genes.

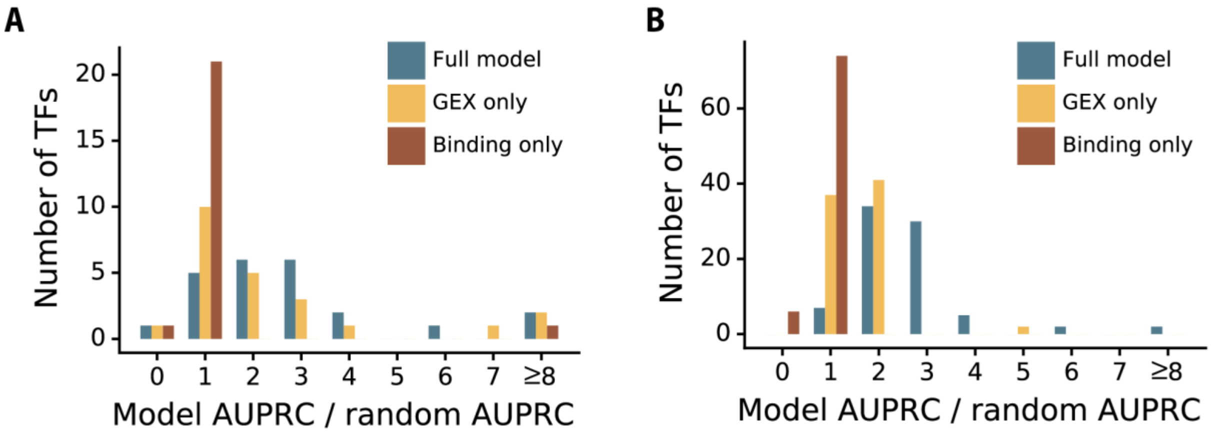

Figure S11. (A) Comparison of full model, GEX only, and binding only using ratio to random for H1 cells. (B) Same as (A) except for HEK293 cells.

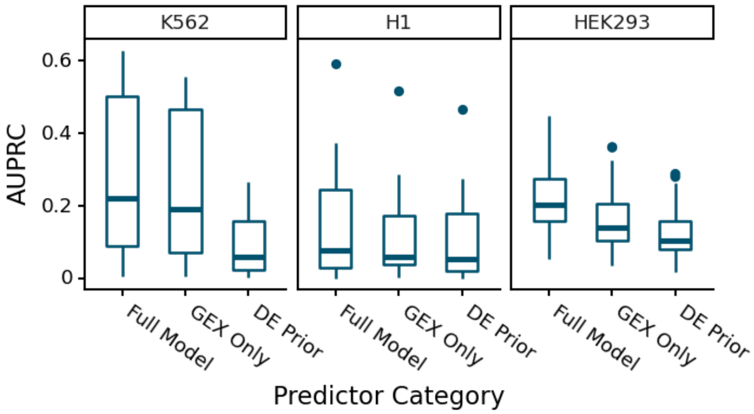

Figure S12. Comparison of model accuracy trained on three categories of predictor variables: the complete set described previously (Full model), only the gene expression features (GEX only), and DE Prior from Crow et al. (2019).

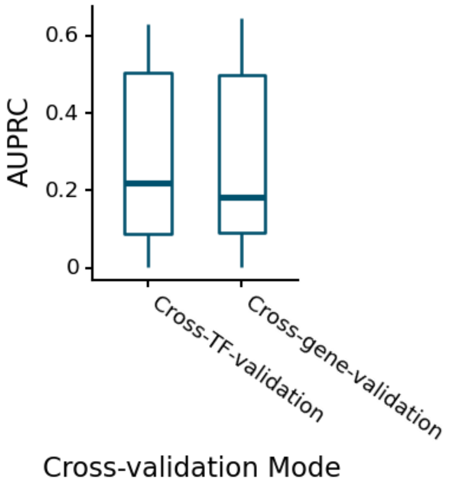

Figure S13. Comparison of predictive accuracy on K562 cells when carrying out cross-validation by TF versus cross-validation by gene. The two procedures yield very similar accuracy distributions.
