# Supplementary material for: Predicting which genes will respond to transcription factor perturbations
Source: G3 (Bethesda). 2022 Jun 6;12(8):jkac144. doi: 10.1093/g3journal/jkac144 (PMC9339286; doi:10.1093/g3journal/jkac144)
Supplement: jkac144_Supplemental_Methods [file jkac144_supplemental_methods.pdf]

Data preparation

TF-perturbation response data

For yeast, we downloaded the microarray data for transcriptional responses 15 minutes after inductions of 194 TF using the ZEV induction system (Hackett et al. 2020). Column *log2_shrunken_timecourses* from the file "Raw & processed gene expression data" at https://idea.research.calicolabs.com/data was used as the levels of responses. Since these values were already shrunken towards zero during analysis, any gene with a non-zero value was defined as responsive.

For human K562 cells, we used all RNA-seq expression profiles measured after gene knockout (KO) or knockdown (KD) in K562 cells from the ENCODE Project database (Dunham et al. 2012; Davis et al. 2018; Abascal et al. 2020). TFKO and TFKD mechanisms include TF-disabling mutations introduced by CRISPR, CRISPR inference (CRISPRi), small-interfering RNA (siRNA), and small-hairpin RNA (shRNA). We downloaded the expected counts of experimental and control profiles that were estimated using RSEM in the ENCODE RNA-seq pipeline and genome assembly GENCODE V24 (GRCh38). For each of the 355 experiments, we ran DESeq2 (V1.10.1) (Love et al. 2014) to identify differentially expressed genes by comparing the experimental replicates to the corresponding control replicates. Genes with Benjamini-Hochberg adjusted P-value < 0.05 and log2 fold-change > 0.5 were considered responsive.

For human H1 (hESC) cells, we used all RNA-seq reads downloaded from NCBI bioproject PRJDB5361 (Nakatake et al. 2020). We ran Nextflow's nf-core/rnaseq to get RSEM expected counts for experimental and control samples for which we had binding data. We ran DESeq2 (Love et al. 2014) to identify differentially expressed genes by comparing the experimental replicates to the corresponding control replicates. Genes with adjusted $P < 0.05$ and absolute log2 fold-change > 0.5 were considered responsive.

For human HEK293 cells, we used all RNA-seq profiles measured after overexpression of each of 80 C2H2 zinc finger transcription factors. Specifically, we downloaded the processed overexpression data from NCBI GEO Series GSE76495 (Schmitges et al. 2016)(file GSE76495_OE .vsd_normalized.log2.txt.gz). Each profile represents the magnitude of differential expression as the profile was batch normalized by subtracting it to the median value of each sequencing batch in log space. Due to the lack of replicates, there was no statistical significance to be calculated; therefore, genes with absolute log2 fold-change > 0.5 were considered responsive.

Pre-perturbation gene expression data

The pre-perturbation expression level feature is the median gene expression level across all samples measured prior to the TF perturbation. For K562 and H1 (RNA-Seq data), we used the log TPM levels among all replicates of control samples from the respective perturbation projects (Dunham et al. 2012; Davis et al. 2018; Abascal et al. 2020; Nakatake et al. 2020). For HEK293, due to the lack of control data, we downloaded RNA-seq profiles of wildtype replicates from NCBI GEO Series GSE122425 (Sun et al. 2019). We removed genes with coefficient of variation > 0.3 because of their extremely low read count. We then calculated the median log TPM levels of all replicates. For yeast (microarray data) we used log fluorescence levels of the red (experimental) channel measured at time 0 (before each of the TF inductions).

To construct a gene expression variation feature that is independent of the expression level, we used the method of (Sigalova et al. 2020). First, we computed the coefficient of variation (COV) of expression level in pre-perturbation samples for each gene. Next, we plotted all genes' COV against their median expression level and fit a smooth curve using locally estimated scatterplot smoothing (LOESS) regression (Python scikit-misc V0.1.3). Each gene's detrended expression variation feature was the residual of its COV from the LOESS fit (Supplemental Fig. S2: right panels).

TF binding location data

All coordinate-dependent features were mapped to yeast genome build sacCer3 and human build GRCh38. Yeast binding location data were generated using transposon calling cards (Wang et al. 2011; Shively et al. 2019; Kang et al. 2020) or ChIP-exo (Bergenholm et al. 2018; Holland et al. 2019). For the calling cards data (16 TFs) we lifted over the transposon insertion coordinates, which were originally mapped based on sacCer2, to sacCer3 using the LiftOver tool in UCSC genome browser. And we considered a target is significantly bound if $P \leq 0.001$ reported in the publications. The binding locations and strengths of ChIP-exo peaks for 20 yeast TFs were obtained directly from the authors of Bergenholm et al. (2018) and Holland et al. (2019). Kang et al. (2020) reported that among the four environmental conditions, the bound targets of these TFs in glucose limited chemostat condition have the best agreement with the perturbation-responsive targets at 15 minutes after TF induction, so we focused on this condition. A peak defined by the authors was required to have signal to noise ratio > 2. Peak locations were reported for the genome of yeast strain CEN.PK, so we lifted them over to coordinates in sacCer3 (strain S288C) as follows. First, since the loci of TFBSs were reported as relative distances to CEN.PK TSSs, these loci were converted to the relative distances to the ORFs using the CEN.PK TSS annotation (https://github.com/SysBioChalmers/ChIPexo_Pipeline/blob/master/Data/TSSData.tsv). Due to high similarity of the two yeast strains, we assumed that the relative distance of each TFBS to CEN.PK ORF are the same for the matching S288C ORF. Next, the relative distances were converted to absolute genomic coordinates in sacCer3 using S288C gene annotation from the *Saccharomyces* Genome Database (SGD).

We downloaded ChIP-seq peaks for 42 TFs in K562 cells and 23 TFs in H1 cells from the ENCODE Project (Dunham et al. 2012; Davis et al. 2018; Abascal et al. 2020). All peaks were mapped to GRCh38 to allow their proper assignment to regulatory regions. We only consider a gene to be a TF if has well-defined DNA-binding factors according to ref. (Lambert et al. 2018). We used only the "conservative" peaks, which had Irreproducible Discovery Rate <= 2%. The log10 q-value reported for each peak was used as the binding signal feature. For HEK293 cells we downloaded the ChIP-seq peaks from NCBI GEO series GSE76494 (file GSE76494_combined_summits.motif_hits.per_protein.hg19.tar.gz) (Schmitges et al. 2016). The genomic coordinates were then converted to GRCh38 using the LiftOver tool in UCSC genome browser.

Histone modifications and chromatin accessibility data

For yeast, we used histone modification data harvested prior to the addition of a diamide stress from ref. (Weiner et al. 2015), which was produced using MNase-ChIP-Seq (GEO accession

GSE61888, https://www.ncbi.nlm.nih.gov/geo/). We used chromatin accessibility data at harvested prior to the introduction of osmotic stress from ref. (Schep et al. 2015) (GSE66386). The coordinates of all yeast genomic features were mapped to sacCer3. For each of the three human cells, we downloaded the coverage data (fold change over control) for histone modifications and chromatin accessibility for the respective cell type from ENCODE (Dunham et al. 2012; Davis et al. 2018; Abascal et al. 2020). And we supplemented the coverage data for H3K27me3 in HEK293 cells using NCBI GEO dataset GSM3907592 (Lamb et al. 2019) due to its unavailability in ENCODE. The coordinates of all human genomic features were mapped to GRCh38.

Mapping genome-wide features to cis-regulatory regions

We defined yeast promoter regions as 1,000 bp upstream to 500 bp downstream from the transcription start site (TSS). TSS coordinates were obtained from ref. (de Boer et al. 2020). Inputs for each genome-wide feature were mapped relative to the TSS and summed over each of 15 100-bp-bins to create 15 features.

For the human cells, TSS coordinates in GRCh38 were downloaded from Ensembl Release 92 (Cunningham et al. 2019). For each gene, we defined the *5' promoter* to be 4 Kb centered on the 5'-end TSS, *alternative promoters* to be 4 Kb region centered at any TSS that is more than 2 Kb from the 5'-end TSS, and *enhancers* to be enhancers that are linked to the gene in the GeneHancer V4.8 database (Fishilevich et al. 2017). We used only "double elite" enhancers, meaning that the enhancers existence and linkage to the target gene are both supported by at least two distinct types of evidence. Enhancers that are more than 500 Kb from the 5'-end TSS were removed.

Each gene must have an equal number of features to create a rectangular feature matrix, even though genes differ greatly in how many alternative TSSs and enhancers they have. For TSSs, we primarily focused on 5'-end, for two reasons. First, approximately half of all alternative TSSs fall within the 2 Kb region downstream from the 5'-end TSS (Supplemental Fig. S1A). The promoter regions of these nearby alternative TSSs largely overlap with that of the 5' TSS, so we did not think it necessary to create separate features for them. Second, the 5' TSS and others within 2 kb of it account for approximately three times as much transcription as the TSSs usage outside the region, according to Fantom5 CAGE data (Forrest et al. 2014; Lizio et al. 2019) (Supplemental Fig. S1B). Alternative promoters outside of this region (median distance 26 kb) were treated as enhancers, since enhancers and promoters share most of their properties and functions (Andersson and Sandelin 2020). Signals located in enhancers were aggregated into features in two ways. The first method (*Prom + bin enhan*; Fig. S3, blue) sums signals within each of 32 bins upstream of the 5' end promoter region and another 32 bins downstream. The width of the bin closest to the TSS was 1 Kb and each subsequent bin was larger by 1 Kb. For example, the widths of the three bins closest to the TSS are 1 Kb, 2 Kb, and 3 Kb. Together, the 32 bins on each side of the promoter covered 498 Kb (-500 Kb to -2 Kb or +2 Kb to +500 Kb). The second method (*Prom + agg enhan*, Fig. S3, green) sums enhancer signals 500 Kb upstream or downstream of the promoter into two features. Note that only signals that fell within defined enhancers were used.

Predicting TF-perturbation responses using cross-validation

For each cell type, we trained and tested a model for predicting whether a gene will respond to the perturbation of TFs by cross validating across all TFs. Each instance is a pair of protein coding gene and TF. In a cross-validation fold, 90% of the TFs were selected at random; therefore, a model was trained to predict the responsiveness of every gene under the perturbation of each selected TF using the genomic and genetic features associated with the gene (e.g., binding signals of the corresponding TF, gene expression features in unperturbed conditions). To assess the model performance, the model was then tested against the held-out TFs. We trained the models using gradient boosted trees implemented in XGBoost library (V 0.90) (Chen and Guestrin 2016). We set the learning rate (eta) to 0.01, the minimum loss reduction at a tree node (gamma) to 5, the subsampling rate on features to 0.8, and subsampling rate on training instances to 0.8. To account for the different number of instances for yeast and human cells, we set the number of gradient boosted trees to 500 for yeast models and 2,500 for human models. In each cross-validation fold, training data were transformed to Z-scores and test data were consequently transformed by using the scaling factors learned from training data. Precision-recall curves were calculated by comparing the predicted probability of response to the true categorization of genes as responsive or not responsive.

Using SHAP to quantify the influences of features on predictions

We employed the SHAP (SHapley Additive exPlanations) framework (V0.35.0) (Lundberg and Lee 2017; Lundberg et al. 2018) to quantify the extent to which each feature contributes to the predicted probability of responsiveness for each gene (TreeExplainer function). SHAP uses a linear model to approximate the predictions for artificially constructed instances in the neighborhood of each actual instance. SHAP values explain why one particular prediction – one TF-gene pair – differs from the average prediction for all genes in response to perturbation of that TF. Positive values indicate how strongly a particular feature value pushes the model toward assigning the gene a higher probability of responding, while negative values represent how strongly the value pushes the model toward assigning the gene a lower probability of responding.

Aggregating SHAP values across genes sets

To characterize the effect of a particular feature for a set of genes, we separately summed its positive and negative SHAP values over genomic bins for each gene. We then averaged the positive sums over all genes and, separately, the negative sums. Formally, we calculated mean positive SHAP value S_k^+ and mean negative SHAP value S_k^- as:

$$S_k^+ = \sum_{i \in G'} \sum_{j \in B} \phi_{ijk} \, \mathbb{1}_{(0,+\infty)}(\phi_{ijk}) / |G'|$$

$$S_k^- = \sum_{i \in G'} \sum_{j \in B} \phi_{ijk} \, \mathbb{1}_{(-\infty,0)}(\phi_{ijk}) / |G'|$$

where ϕ_{ijk} is the SHAP value for gene i in bin j for feature k, G' is the set of gene indices, ($G' \subseteq \mathrm{G}$, where G is for all genes), and B is the set of bin indices. For coordinate-independent features (e.g., pre-perturbation gene expression), B has size of one. We defined the *Net influence* of a feature on predictions for a set of genes as a single sum including both positive and negative SHAP values of the feature over the set of genes. *Net influence* measures the feature's overall direction of influence. *Global feature importance* is the sum of absolute values of the SHAP values of a feature for genes in the set. *Global feature importance* measures how important the feature is in determining the model's prediction, regardless of direction.

Calculating the P-values using randomized permutation experiments

We trained 35 randomly permuted models for the Calling Cards, ChIP-exo, H1, and K562 datasets. We trained 5 randomly permuted models for the HEK293 dataset due to time and memory constraints. We predicted the log10 standard deviations with OLS using the log10 SD of the permuted model AUPRCs for TFs which had random expectation > 0.01 (log10 random expectation > -2). For TFs with random expectation > 0.01, we used the SD on the fitted line to calculate the Z-score; for TFs with random expectation ≤ 0.01, we used the permuted AUPRC SD. We then calculated a P-value in an upper-tail hypothesis test using these Z-scores. To account for the fewer randomly permuted models for the HEK293 datatset, we regressed over the top SD values in each quintile, binning by random expectation.

REFERENCES

Abascal F, Acosta R, Addleman NJ, Adrian J, Afzal V, Aken B, Akiyama JA, Jammal O Al, Amrhein H, Anderson SM, et al. 2020. Expanded encyclopaedias of DNA elements in the human and mouse genomes. Nature. 583(7818):699–710. doi:10.1038/s41586-020-2493-4.

Andersson R, Sandelin A. 2020. Determinants of enhancer and promoter activities of regulatory elements. Nat Rev Genet. 21(2):71–87. doi:10.1038/s41576-019-0173-8. http://dx.doi.org/10.1038/s41576-019-0173-8.

Bergenholm D, Liu G, Holland P, Nielsen J. 2018. Reconstruction of a Global Transcriptional Regulatory Network for Control of Lipid Metabolism in Yeast by Using Chromatin Immunoprecipitation with Lambda Exonuclease Digestion. mSystems. doi:10.1128/msystems.00215-17.

de Boer CG, Vaishnav ED, Sadeh R, Abeyta EL, Friedman N, Regev A. 2020. Deciphering eukaryotic gene-regulatory logic with 100 million random promoters. Nat Biotechnol. 38(1):56–65. doi:10.1038/s41587-019-0315-8. http://dx.doi.org/10.1038/s41587-019-0315-8.

Chen T, Guestrin C. 2016. XGBoost: A scalable tree boosting system. In: Proceedings of the ACM SIGKDD International Conference on Knowledge Discovery and Data Mining.

Cunningham F, Achuthan P, Akanni W, Allen J, Amode MR, Armean IM, Bennett R, Bhai J, Billis K, Boddu S, et al. 2019. Ensembl 2019. Nucleic Acids Res. doi:10.1093/nar/gky1113.

Davis CA, Hitz BC, Sloan CA, Chan ET, Davidson JM, Gabdank I, Hilton JA, Jain K, Baymuradov UK, Narayanan AK, et al. 2018. The Encyclopedia of DNA elements (ENCODE): Data portal update. Nucleic Acids Res. doi:10.1093/nar/gkx1081.

Dunham I, Kundaje A, Aldred SF, Collins PJ, Davis CA, Doyle F, Epstein CB, Frietze S, Harrow J, Kaul R, et al. 2012. An integrated encyclopedia of DNA elements in the human genome. Nature. doi:10.1038/nature11247.

Fishilevich S, Nudel R, Rappaport N, Hadar R, Plaschkes I, Iny Stein T, Rosen N, Kohn A, Twik M, Safran M, et al. 2017. GeneHancer: genome-wide integration of enhancers and target genes in GeneCards. Database. 2017(1):1–17. doi:10.1093/database/bax028. https://academic.oup.com/database/article-lookup/doi/10.1093/database/bax028.

Forrest ARR, Kawaji H, Rehli M, Baillie JK, De Hoon MJL, Haberle V, Lassmann T, Kulakovskiy I V., Lizio M, Itoh M, et al. 2014. A promoter-level mammalian expression atlas. Nature. 507(7493):462–470. doi:10.1038/nature13182.

Hackett SR, Baltz EA, Coram M, Wranik BJ, Kim G, Baker A, Fan M, Hendrickson DG, Berndl M, Mcisaac RS. 2020. Learning causal networks using inducible transcription factors and transcriptome-wide time series. :1–15. doi:10.15252/msb.20199174.

Holland P, Bergenholm D, Börlin CS, Liu G, Nielsen J. 2019. Predictive models of eukaryotic transcriptional regulation reveals changes in transcription factor roles and promoter usage between metabolic conditions. Nucleic Acids Res. doi:10.1093/nar/gkz253.

Kang Y, Patel NR, Shively C, Recio PS, Chen X, Wranik BJ, Kim G, McIsaac RS, Mitra R, Brent MR. 2020. Dual threshold optimization and network inference reveal convergent evidence from TF binding locations and TF perturbation responses. Genome Res.:gr.259655.119. doi:10.1101/gr.259655.119.

Lamb KN, Bsteh D, Dishman SN, Moussa HF, Fan H, Stuckey JI, Norris JL, Cholensky SH, Li

D, Wang J, et al. 2019. Discovery and Characterization of a Cellular Potent Positive Allosteric Modulator of the Polycomb Repressive Complex 1 Chromodomain, CBX7. Cell Chem Biol. 26(10). doi:10.1016/j.chembiol.2019.07.013.

Lambert SA, Jolma A, Campitelli LF, Das PK, Yin Y, Albu M, Chen X, Taipale J, Hughes TR, Weirauch MT. 2018. The Human Transcription Factors. Cell. 172(4):650–665. doi:10.1016/j.cell.2018.01.029. https://doi.org/10.1016/j.cell.2018.01.029.

Lizio M, Abugessaisa I, Noguchi S, Kondo A, Hasegawa A, Hon CC, De Hoon M, Severin J, Oki S, Hayashizaki Y, et al. 2019. Update of the FANTOM web resource: Expansion to provide additional transcriptome atlases. Nucleic Acids Res. doi:10.1093/nar/gky1099.

Love MI, Huber W, Anders S. 2014. Moderated estimation of fold change and dispersion for RNA-seq data with DESeq2. Genome Biol. 15(12):1–21. doi:10.1186/s13059-014-0550-8.

Lundberg S, Lee S-I. 2017. A Unified Approach to Interpreting Model Predictions. NIPS. 16(3):426–430.

Lundberg SM, Erion GG, Lee S. 2018. Consistent Individualized Feature Attribution for Tree Ensembles. (2). http://arxiv.org/abs/1802.03888.

Nakatake Y, Ko SBH, Sharov AA, Wakabayashi S, Murakami M, Sakota M, Chikazawa N, Ookura C, Sato S, Ito N, et al. 2020. Generation and Profiling of 2,135 Human ESC Lines for the Systematic Analyses of Cell States Perturbed by Inducing Single Transcription Factors. Cell Rep. 31(7):107655. doi:10.1016/j.celrep.2020.107655. https://doi.org/10.1016/j.celrep.2020.107655.

Schep AN, Buenrostro JD, Denny SK, Schwartz K, Sherlock G, Greenleaf WJ. 2015. Structured nucleosome fingerprints enable high-resolution mapping of chromatin architecture within regulatory regions. Genome Res. doi:10.1101/gr.192294.115.

Schmitges FW, Radovani E, Najafabadi HS, Barazandeh M, Campitelli LF, Yin Y, Jolma A, Zhong G, Guo H, Kanagalingam T, et al. 2016. Multiparameter functional diversity of human C2H2 zinc finger proteins. Genome Res. doi:10.1101/gr.209643.116.

Shively CA, Liu J, Chen X, Loell K, Mitra RD. 2019. Homotypic cooperativity and collective binding are determinants of bHLH specificity and function. Proc Natl Acad Sci U S A. doi:10.1073/pnas.1818015116.

Sigalova O, Shaeiri A, Forneris M, Furlong E, Zaugg J. 2020. Predictive features of gene expression variation reveal a mechanistic link between expression variation and differential expression. :1–24. doi:10.1101/2020.02.10.942276.

Sun Z, Xue S, Xu H, Hu X, Chen S, Yang Z, Yang Y, Ouyang J, Cui H. 2019. Effects of NSUN2 deficiency on the mRNA 5-methylcytosine modification and gene expression profile in HEK293 cells. Epigenomics. 11(4). doi:10.2217/epi-2018-0169.

Wang H, Mayhew D, Chen X, Johnston M, Mitra RD. 2011. Calling Cards enable multiplexed identification of the genomic targets of DNA-binding proteins. Genome Res. doi:10.1101/gr.114850.110.

Weiner A, Hsieh TS, Rando OJ, Friedman N, Weiner A, Hsieh TS, Appleboim A, Chen H V, Rahat A, Amit I. 2015. High-Resolution Chromatin Dynamics during a Yeast Resource High-Resolution Chromatin Dynamics during a Yeast Stress Response. Mol Cell. 58(2):371–386. doi:10.1016/j.molcel.2015.02.002. http://dx.doi.org/10.1016/j.molcel.2015.02.002.
